# Characterization of thyrotropin-releasing hormone producing neurons in sea urchin, from larva to juvenile

**DOI:** 10.3389/fnins.2024.1378520

**Published:** 2024-04-10

**Authors:** Maria Cocurullo, Periklis Paganos, Giovanna Benvenuto, Maria Ina Arnone

**Affiliations:** Department of Biology and Evolution of Marine Organisms, Stazione Zoologica Anton Dohrn, Naples, Italy

**Keywords:** TRH, sea urchin, nervous system, neuropeptide, development, neurosecretory cells, invertebrates

## Abstract

Most sea urchin species are indirect developers, going through a larval stage called pluteus. The pluteus possesses its own nervous system, consisting mainly of the apical organ neurons (controlling metamorphosis and settlement) and ciliary band neurons (controlling swimming behavior and food collection). Additional neurons are located in various areas of the gut. In recent years, the molecular complexity of this apparently “simple” nervous system has become apparent, with at least 12 neuronal populations identified through scRNA-sequencing in the species *Strongylocentrotus purpuratus*. Among these, there is a cluster of neurosecretory cells that produce a thyrotropin-releasing hormone-type neuropeptide (TRHergic) and that are also photosensory (expressing a Go-Opsin). However, much less is known about the organization of the nervous system in other sea urchin species. The aim of this work was to thoroughly characterize the localization of the TRHergic cells from early pluteus to juvenile stages in the Mediterranean sea urchin species *Paracentrotus lividus* combining immunostaining and whole mount *in situ* hybridization. We also compared the localization of TRHergic cells in early plutei of two other sea urchin species, *Arbacia lixula* and *Heliocidaris tuberculata*. This work provides new information on the anatomy and development of the nervous system in sea urchins. Moreover, by comparing the molecular signature of the TRHergic cells in *P. lividus* and *S. purpuratus*, we have obtained new insights how TRH-type neuropeptide signaling evolved in relatively closely related species.

## 1 Introduction

Thyrotropin-Releasing Hormone (TRH) is a short neuropeptide comprising just three amino-acid residues in vertebrates (pGlu-His-Pro-NH2) which is mainly known to have a central role in regulating the Hypothalamus-Pituitary-Thyroid Axis in mammals, therefore controlling a variety of functions from metabolism and food intake to growth and reproduction. Due to this central function, an extensive literature exists focusing on rats and mice; for reviews see Nillni (2010, 2018) and Lechan et al. (2018).

TRH signaling activity is mediated by specific TRH receptors (TRHR) which belong to the G protein-coupled receptors (GPCRs) family (for a review see Galas et al., 2009).

While the main function of TRH as a regulator of metabolism and food intake is conserved in vertebrates, the TRH downstream pathway seems to have been rewired, as also indicated by the species-specificity of TRH and TRHR localizations. A very good review on this topic can be found in Galas et al. (2009).

Interestingly, in vertebrates the TRH function is not only limited to its hypophysiotropic role. Indeed, the wide distribution of TRH and TRHR transcripts across the brain suggests a function as neurotransmitter or neuromodulator (Sharif, 1989; Calzá et al., 1992; Heuer et al., 2000). Extra-hypothalamic and non-hypophysiotropic TRH distribution and function have been reviewed in Morley (1979) and Lechan et al. (2018).

Despite the extensive literature addressing the role of TRH in vertebrates, much less is known in invertebrates. Only in 2017, functional analysis of TRH-type signaling in *C. elegans* suggested that the ancestral role of TRH-type neuropeptides was the control of postembryonic growth and reproduction (Van Sinay et al., 2017). Meanwhile, short EFLa/EFLGa peptides have been identified in insects and annelids. However, their homology with TRH-type neuropeptides has been suggested only by deorphanization experiments showing that a EFLGa peptide is cognate ligand of the *Platynereis dumerilii* TRHR (Bauknecht and Jékely, 2015). However, the function of EFLa/TRH remains still elusive (Kotwica-Rolinska et al., 2020; Veenstra and Šimo, 2020). In conclusion, despite the numerous evidence that EFLa/TRH is an evolutionary ancient signal, originating before the divergence of protostomes and deuterostomes, we know very little about its ancestral function(s) and evolution (Bauknecht and Jékely, 2015; Van Sinay et al., 2017; Elphick et al., 2018). In this perspective, echinoderms such as sea urchins represent a key group of organisms to study how not only the TRH pathway but the whole neurosecretory nervous system evolved (Semmens and Elphick, 2017). In fact, as deuterostome invertebrates belonging to the phylum Echinodermata, sea urchins fill the gap between vertebrates and protostomian invertebrate models such as *C. elegans*, *Drosophila* and *P. dumerilii*.

**Figure 1:**
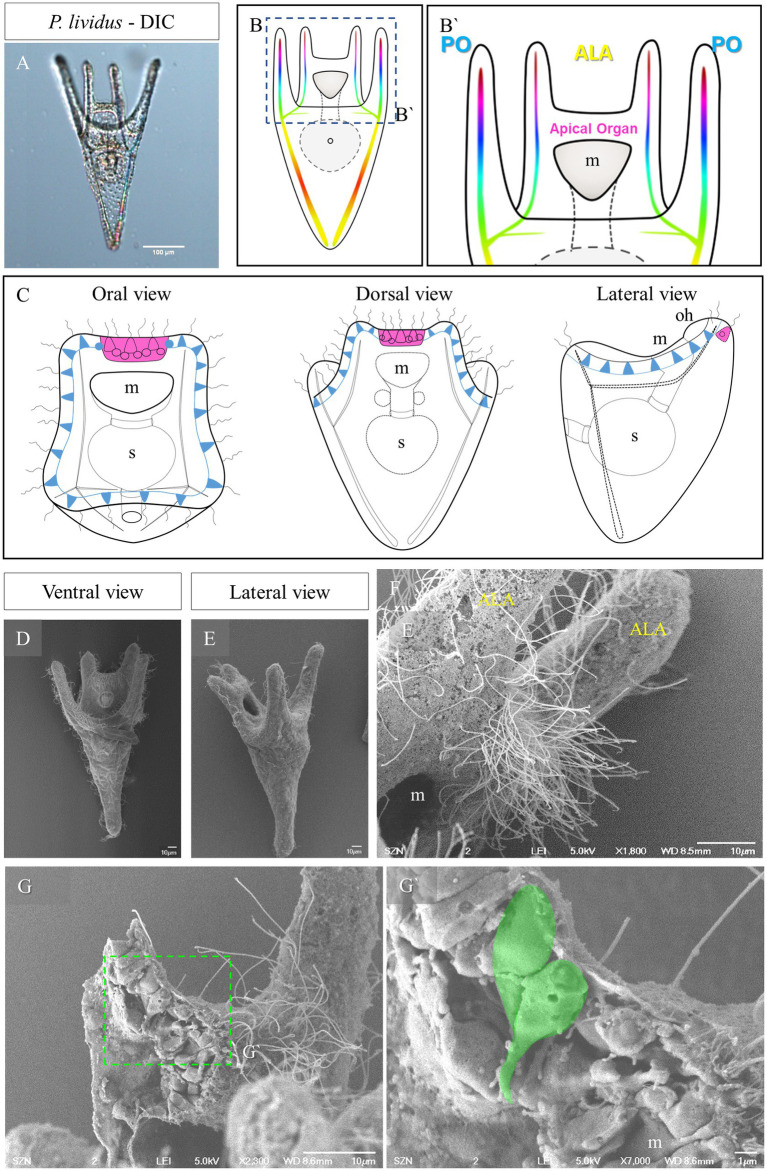
Morphology of *P. lividus* larvae at pluteus stage. **(A)** A 4-armed *P. lividus* pluteus (72 h post fertilization, hpf) imaged using Differential Interference Contrast (DIC) microscopy. Anterolaretal arm, ALA, in yellow. Post oral arm, PO, in cyan. **(B)** Schematic representation of a *P. lividus* the 4-armed pluteus larva. **(B′)** Detail of the area squared in **(B)** showing the localization of the apical organ. **(C)** A schematic representation of an early pluteus and its nervous system shown from three different views: in magenta is highlighted the apical organ, in light blue are highlighted the neurons that run underneath the ciliary band. **(D–F)** Larva at 72 hpf imaged at SEM. Panel **(D)** is a ventral view as illustrated in **(B)**. Panel **(E)** is a lateral view as illustrated in **(C)**. In F, there is a close up on the apical organ area from a lateral view, showing that the epithelium is densely ciliated. **(G,G’)** A freeze-fractured larva imaged using SEM shows the morphology of the cells in the apical plate; in **(G’)**, a close up on two cells, highlighted in green, showing a flask-shape morphology. m, mouth; s, stomach; oh, oral hood; scale bars: **(A)** = 100 μm, **(D–G)** = 10 μm, **(G’)** = 1 μm.

Sea urchins have a complex life cycle and most species display indirect development. In these cases, the first developmental stages do not directly give rise to an adult individual, but produces a planktotrophic larva, called pluteus, capable of free swimming and feeding (for a detailed description of the early larva morphology see Figure 1). The sea urchin pluteus spends from few weeks to months in the water column feeding on unicellular algae, and eventually goes through metamorphosis (Giudice, 1986; Schroeder, 1986; Ettensohn et al., 2004). Nonetheless, examples of sea urchin lecithotrophic larvae, displaying direct development, also exist, even in the context of the same genus (Raff, 1992), giving the opportunity to compare the two life cycle forms in closely related species (Byrne et al., 2001; Katow et al., 2009). The morphology and development of planktotrophic sea urchins have been described in details from fertilization to metamorphosis by, among others (Smith et al., 2008; Formery et al., 2022).

In general, sea urchins are considered to have a rather simple nervous system, especially at the larva stage, displaying only basic responses to the environment. The larval nervous system can be divided in a “central nervous system” and in a “peripheral nervous system” (Burke et al., 2006a,b). The central nervous system consists mainly of a group of serotonergic neurons positioned in the apical organ. These neurons are ciliated cells and their function has been linked with metamorphosis (Hadfield et al., 2016), ciliary beating (Yoshihiro et al., 1992; Voronezhskaya and Khabarova, 2003; Yaguchi and Katow, 2003), and pyloric sphincter opening in response to light (Yaguchi and Yaguchi, 2021). For a review on the apical organ structure in echinoderms see (Byrne et al., 2007). The organization of the serotonergic neurons is very peculiar, appearing as huge flask shaped serotonergic cells interspersed by smaller interneurons having a spherical shape (Nakajima et al., 2004). These cells are localized just above the mouth, between the anterolateral arms (Bisgrove and Burke, 1986; Beer et al., 2001).

The larval peripheral nervous system consists of a set of neurons innervating the ciliary band, a pair of ganglia situated on the lower lip of the mouth, and additional neuronal cells associated with the gut. From these cells, nerve fibers project to connect neurons and to innervate the aboral epithelium (Burke, 1978; Bisgrove and Burke, 1987). Despite the apparent simplicity of the sea urchin nervous system, the analysis of the *Strongylocentrotus purpuratus* genome first revealed the complexity of the neuropeptidergic system leading to the identification of about 40 genes encoding neuropeptide precursors (Rowe and Elphick, 2012), including also a *Sp-Trh* (SPU_008352). Moreover, recent analysis contributed to unravel a previously unknown complexity at the neurochemical level: in particular Paganos and colleagues, reconstructing the larval cell type families at a single cell resolution, were able to identify up to 12 neuronal types at early pluteus stage (likely representing different cell types), expanding our understanding of the complexity of the sea urchin larva nervous system (Paganos et al., 2021). Regarding the sea urchin TRHergic cells, we still know very little. From previous work we know that these cells arise at early larva stage in the species *S. purpuratus* as a couple of cells bilaterally distributed at the sides of the larval apical organ (Wood et al., 2018; Paganos et al., 2021; Cocurullo et al., 2023). Moreover, in *S. purpuratus*, these cells are both neurosecretory (producing a variety of signaling molecules such as kisspeptin-type and SALMFamide-type neuropeptides) and sensory (they express the Go-Opsin3.2 and a series of receptors). In particular, in Cocurullo et al. (2023) we focused our attention on the characterization of the photoreceptor function of the TRHergic cells. Intriguing is the evidence that the sea urchin *Hemicentrotus pulcherrimus* larva also displays a couple of photoreceptors expressing the Go-Opsin and these cells have been shown to regulate the pyloric sphincter opening in response to light (Yaguchi and Yaguchi, 2021). However, whether these photoreceptors are TRHergic and whether this cell type is conserved in all sea urchin larvae is still unknown.

The adult sea urchin nervous system primarily consists of a nerve ring encircling the gut on the oral side of the animal and five radial nerves arising from it. Smaller fibers branching from each radial nerve innervate all appendices (spines, tube feet and pedicellariae). Ganglia are present too, especially at the base of the spines and of the disk situated at the most distal part of the tube feet. The adult nervous system is formed *de novo* within the rudiment during larval development and is generally thought to be independent from the larval one (Nakajima et al., 2004; Burke et al., 2006a; Smith et al., 2008), although evidence of a connection between the larva and the rudiment nervous system has been reported in *Hemicentrotus pulcherrimus* (Katow et al., 2009). In conclusion, sea urchins (and Echinoderms in general), represent a unique system to investigate the evolution and function of various features of the nervous system, such as evolution and function of vision (Nilsson, 2013), evolution of neuropeptidergic signaling (Semmens et al., 2016; Semmens and Elphick, 2017), and evolution of organogenesis (Perillo et al., 2018; Paganos et al., 2022b).

In this work we characterized distribution of TRHergic cells in the sea urchin species *Paracentrotus lividus* from early pluteus to juvenile post metamorphosis stages. In contrast with the most studied sea urchin species *S. purpuratus*, in fact, *P. lividus* reaches metamorphosis in a relatively short time (about 1 month, compared to the roughly 3 months required for *S. purpuratus*). A thorough description of *P. lividus* development from fertilization to metamorphosis was recently published by Formery et al. (2022) and includes timing of all the stages. This gives us the possibility to study in detail how the sea urchin nervous system grows during the whole development and how the adult nervous system arises and connects to the larva nervous system. Moreover, because *S. purpuratus* and *P. lividus* are thought to have diverged from a common ancestor relatively recently (~ 40 million years ago), comparison of these species is a powerful approach to understand how sensory and neurosecretory systems have evolved. Finally, we compared the distribution of TRHergic cells in early plutei of the Mediterranean *P. lividus* and the Pacific ocean *S. purpuratus* with those of two more sea urchin species: *Arbacia lixula,* sharing the same habitat of *P. lividus*, and *Heliocidaris tuberculata*, another Pacific Ocean sea urchin species living at temperatures more similar to *P. lividus*. Our findings suggest that there are differences in the development and function of TRHergic cells among sea urchin species.

## 2 Results

The sea urchin species investigated in this study all develop indirectly and produce a planktotrophic larva called a pluteus. The morphology of the pluteus at early larva stage is summarized in Figure 1. They are small (around 200 μm) and transparent (Figure 1A) allowing us to observe inner structures such as the tripartite gut and the skeleton. The morphology of the *P. lividus* larva shown in Figure 1A is illustrated in Figures 1B,B’. At this stage the larva has two pairs or arms, one above the mouth, which are called anterolateral arms (ALA), and two on the ventral side known as post oral arms (PO). Seen from the top (oral view, Figure 1C) we have a clear view of the mouth and of the ALA, and PO arms. Moreover, it is possible to observe the ciliary band neurons (light blue in Figure 1C) and the apical organ (magenta in Figures 1B’,C), localized between the ALA, on the dorsal side. From a lateral view, the larva can be divided in a dorsal and a ventral side. Opposite the oral epithelium that surrounds the mouth, there is the aboral epithelium. Imaged using SEM, the area between the ALA, known as apical plate, appears densely ciliated (Figures 1D–F). When freeze fractured, this area shows a densely packed epithelium in which we observed a couple of cells having a peculiar shape (Figures 1G,G’). These two cells, localized right at the corner between the ALA and the aboral epithelium, have an elongated morphology, with the cell body facing the external environment and an extension of the cell body, which looks like a projection, facing the internal environment.

### 2.1 Identification of TRHergic cells at early larva stage

**Figure 2:**
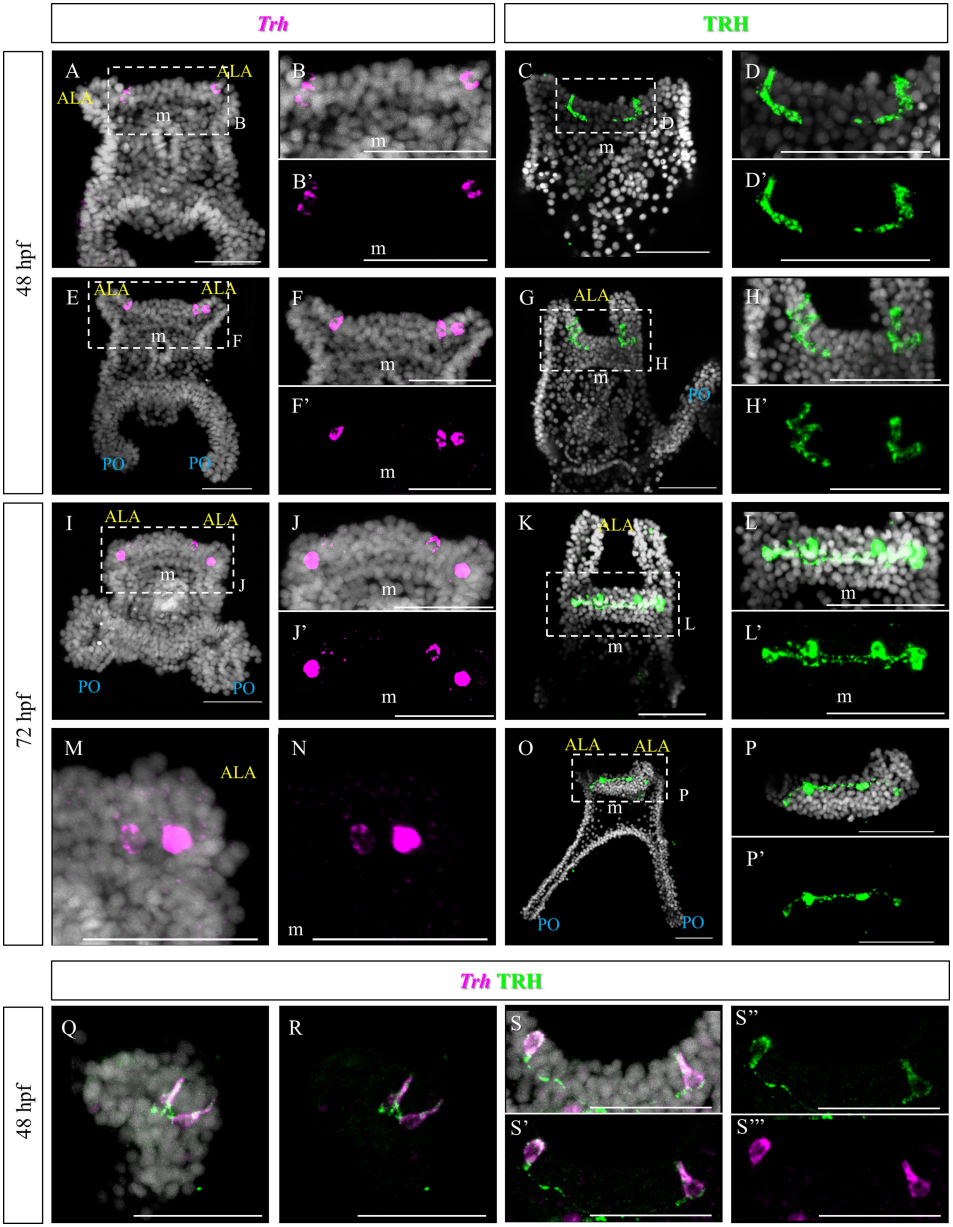
TRHergic cells in *P. lividus* larvae at early pluteus stage. **(A–B’,E–F’)** FISH of 48 hpf larvae showing two *Trh* + cells located at the base of the ALA. **(I–J’,M,N)** FISH of 72 hpf larvae. **(C–D′,G–H’)** IHC of 48 hpf larvae performed using custom made Sp-TRH antibodies, highlighting not only the cell bodies but also some short projections. **(K–L’,O–P’)** IHC performed on 72 hpf larvae reveals four cell bodies, connected by axons. In **(O)** it is shown that the most external cells are located on the oral side, while the internal ones more dorsally. **(Q–S”’)** Double TRH FISH and IHC of 48 hpf larvae show colocalization of *Trh* mRNA and TRH peptide in the cell body, while only TRH peptide was stained in the projections. Nuclei are labeled with DAPI (in white). All pictures are maximum projections of confocal images. m, mouth; s, stomach; oh, oral hood; ALA, anterolateral arm; PO, post oral arm; scale bars = 50 μm.

The TRHergic cells were identified using Whole-Mount Fluorescent *In Situ* Hybridization (FISH) and Immunohistochemistry (IHC). IHC was performed using a custom antibody (Wood, 2020; Cocurullo et al., 2023). The TRHergic cells first arise as one or two cells located right at the connection between the ALA and the apical plate (Figures 2A–H’), where the peculiar shaped cells were observed using SEM (Figure 1G). These cells seem to project axons toward the apical organ plate and show an elongated morphology with IHC (Figures 2C–D’). Their number quickly increases and already at 48 h post fertilization (hpf) it is possible to observe 3 or 4 cells labelled (Figures 2E–H). The localization of these cells is better appreciated by IHC, since during the FISH procedure the larval arms, lacking the support from the skeleton, often collapse (e.g., Figures 2I,M). At 72 hpf, the number of TRHergic cells is stabilized at four, two at the base of the arms, on the dorsal side, and two more external facing the oral side (Figures 2I–P’). Their localization can be fully appreciated in an oral view, like in Figures 2O–P’. A full projection of confocal pictures taken from dorsal or ventral views might suggest that they are localized on the same plane (e.g., Figures 2K–L’). Nonetheless, when only the individual stacks are analyzed it becomes obvious that these groups of cells are not localized on the same focal plane (Supplementary Figure S1). Intriguingly, at 72 hpf, these cells have long projections which colonize the area between the ALA, on the dorsal side of the pluteus, where the apical organ is located. Double FISH and IHC on 48 hpf confirm colocalization of *Trh* mRNA and TRH peptide in the cell body, while TRH peptide only is stained in the cell projections (Figures 2Q–S’’’).

### 2.2 Tracking TRHergic cells during larval development in *Paracentrotus lividus*

To follow the fate of the TRHergic cells during sea urchin larva development, IHC was performed on older larval stages. To describe the morphology of late *P. lividus* developmental stages, we used the detailed developmental atlas produced by Formery et al. (2022).

**Figure 3:**
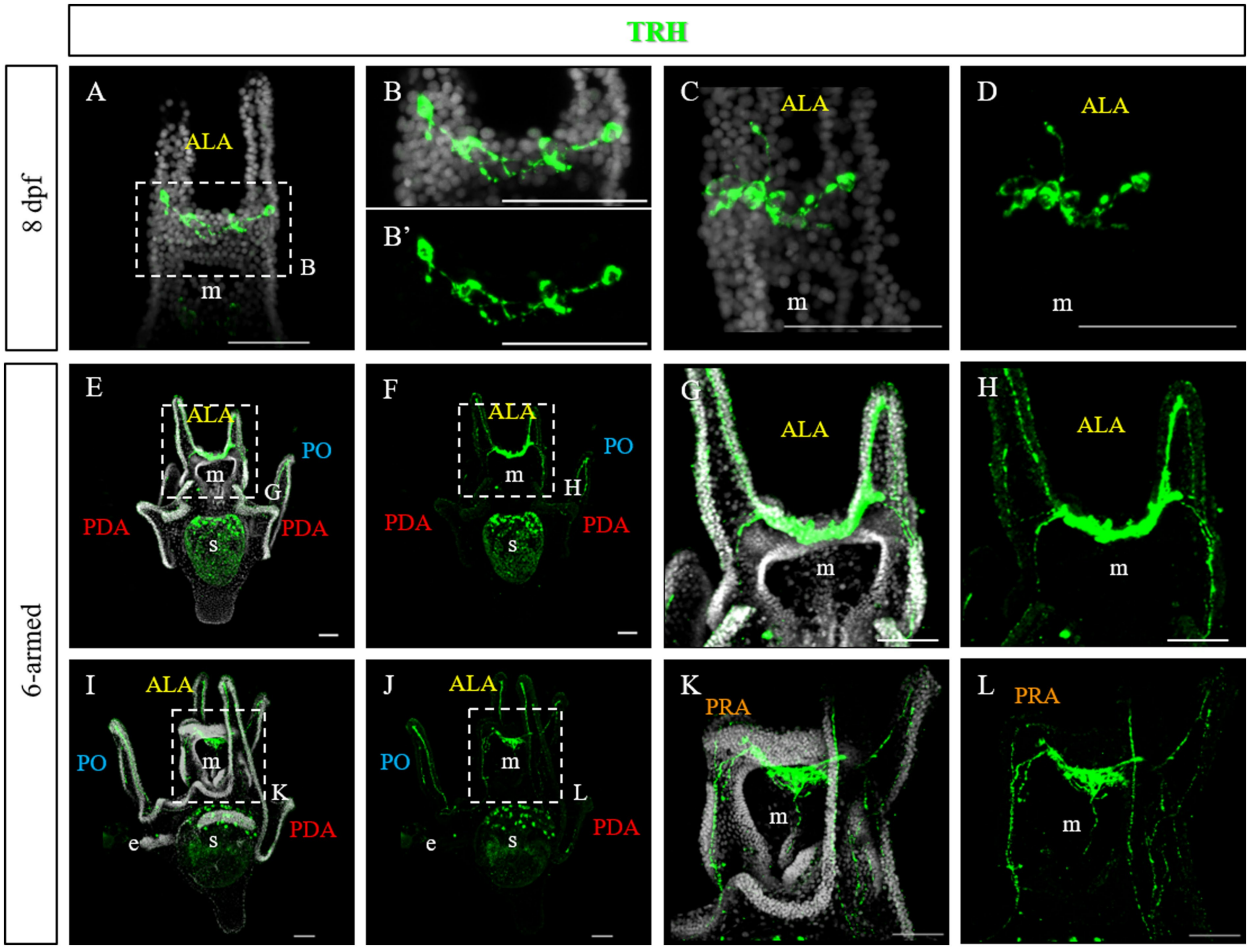
TRHergic cells in *P. lividus* larvae at late pluteus stage. **(A–D)** IHC was performed on 8 dpf larvae. At this stage the network of TRHergic axons is more extended that at early stages, and some projections begin to colonize the PO **(C,D)**. **(E–L)** IHC was performed on 6-armed larvae showing that TRHergic cells and projections colonize the whole oral hood. From the sides of the central ganglia, right at the connection with the ALA, the nerve fiber branches out, part goes underneath the ALA, part goes under the buds, where a second pair of ALA is starting to grow. Nuclei are labeled with DAPI (in white). All pictures are maximum projections of confocal images. m, mouth; s, stomach; oh, oral hood; e, epaulette; ALA, anterolateral arm; PO, post oral arm; PDA, posterodorsal arm; PRA, preoral arm, scale bars = 50 μm.

At late 4-armed pluteus stage, 8 days post fertilization (dpf), the network of TRHergic cells is widely extended, and even some interneurons localized in the apical organ plate appear to be stained (Figures 3A–D). In some cases, TRH positive cells are localized in the ALA. Occasionally, a projection originating from the cell at the corner with the oral ectoderm integrates into the arm region and runs underneath the ciliary band (Figures 3C,D). At the 6-armed pluteus stage, the number of TRHergic cells and the network of connections between them increases (Figures 3E–L), and they colonize the whole apical plate. In particular, while the number of cell bodies observed in single stacks is limited (Supplementary Figure S2), the network of connections between TRHergic cells is extensive and fully colonize the oral hood. In one case, projections from the apical organ extending down the mouth epithelium were also observed (Figures 3I–L and Supplementary Figure S2C). Moreover, from the sides of the apical plate, the projections branch out. A first TRHergic axon runs underneath the ciliary band covering the ALA, goes down the epithelium at the side of the mouth, and innervates also the PO ciliary band, while a second group of axons branches from the apical organ area and innervates the buds of the second pair of ALA that will soon rise, which are called preoral arms or PRA (Figures 3G,H,K,L).

**Figure 4:**
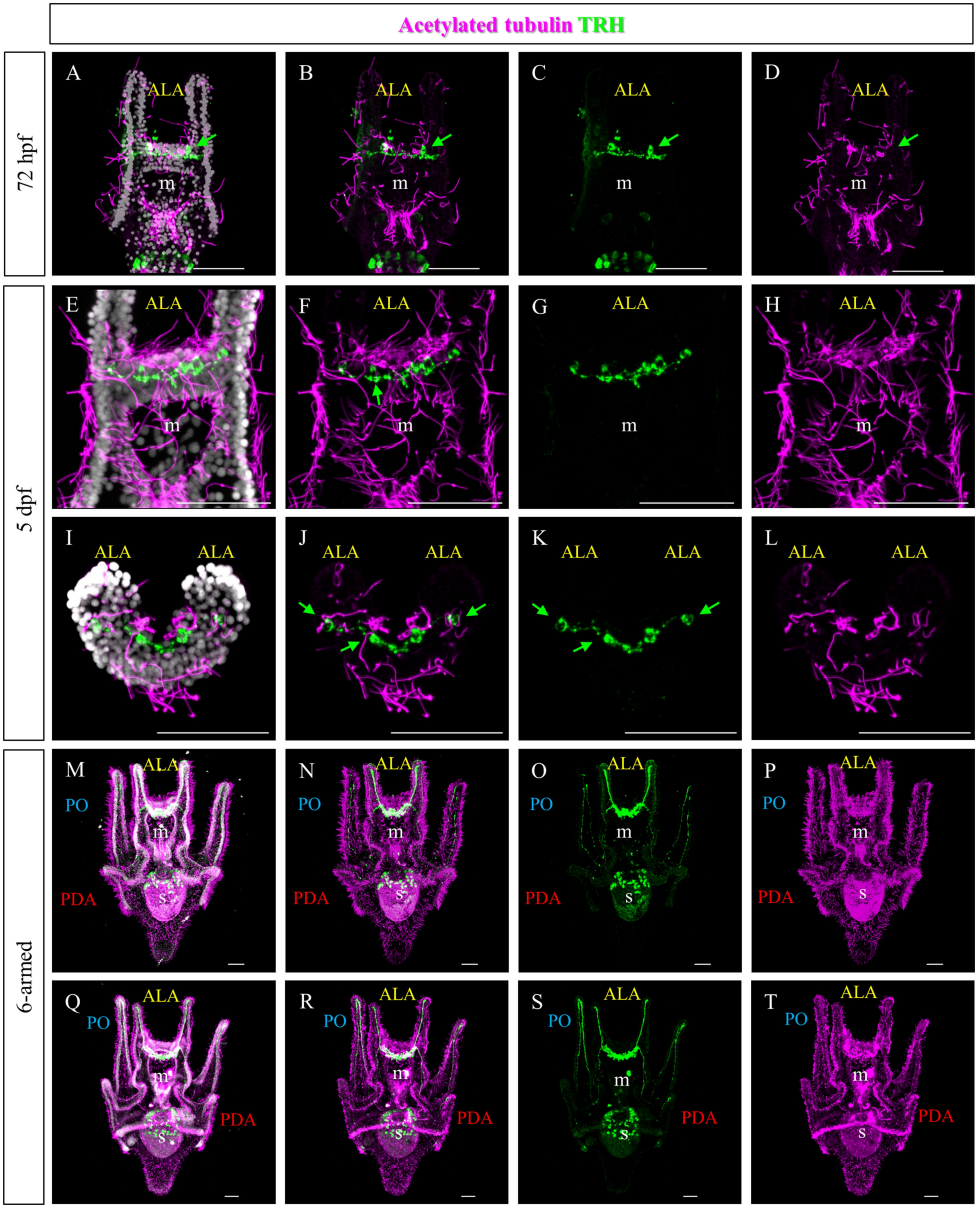
Characterization of the TRHergic cells in relation to the ciliary band across larval development *in P. lividus*. Double IHC for TRH and acetylated tubulin was performed at different stages in *P. lividus*. **(A–D)** A 72 hpf larva showing that the TRHergic cells are ciliated. **(E–H)** A larva at 5 dpf is shown in ventral view. **(I–L)** A 5 dpf larva is shown from an oral view with the dorsal side up and the oral side (which can be recognized thanks to the tuft of cilia in the middle) facing down; only the area above the mouth is visible. From this perspective it is possible to appreciate the fact that the TRHergic cells between the two PO are located on the very back of the larva. **(M–T)** The double IHC at 6-armed pluteus stage highlights the fact that the TRHergic cells colonized the whole oral hood, while the projections run underneath the ciliary band, but nor under the epaulettes. Nuclei are labeled with DAPI (in white). Green arrows highlight the TRHergic cells that are clearly ciliated. All pictures are maximum projections of confocal pictures. m, mouth; s, stomach; oh, oral hood; e, epaulette; ALA, anterolateral arm; PO, post oral arm; PDA, Posterodorsal arm, scale bars = 50 μm.

To highlight the ciliary band, larvae at different stages were stained using an anti-acetylated tubulin antibody. At 72 hpf and 5 days post fertilization (dpf), numerous cilia are localized not only in the ciliary band, but also in the area between the ALA and on the oral hood (Figures 4A,E,I and Figure 1E). Double staining showed that the TRHergic cells are ciliated cells (Figures 4A–L, see green arrows and white arrows in Supplementary Figure S3). In plutei at 6-armed stage, the ciliary band extends on all the arms, and the TRHergic axons run underneath (Figures 4M–P). In older 6–armed larvae, two ciliated structures begin to form below the PO arms, these are called epaulettes and are responsible for larval swimming at later stages (Smith et al., 2008). Intriguingly, the TRHergic axons do not run underneath the epaulettes (Figures 4Q–T and Supplementary Figures S4B,C for a close-up). Moreover, no TRHergic axons connect to the rudiment that is forming on the left side of the larva (Supplementary Figures S4D,E).

**Figure 5:**
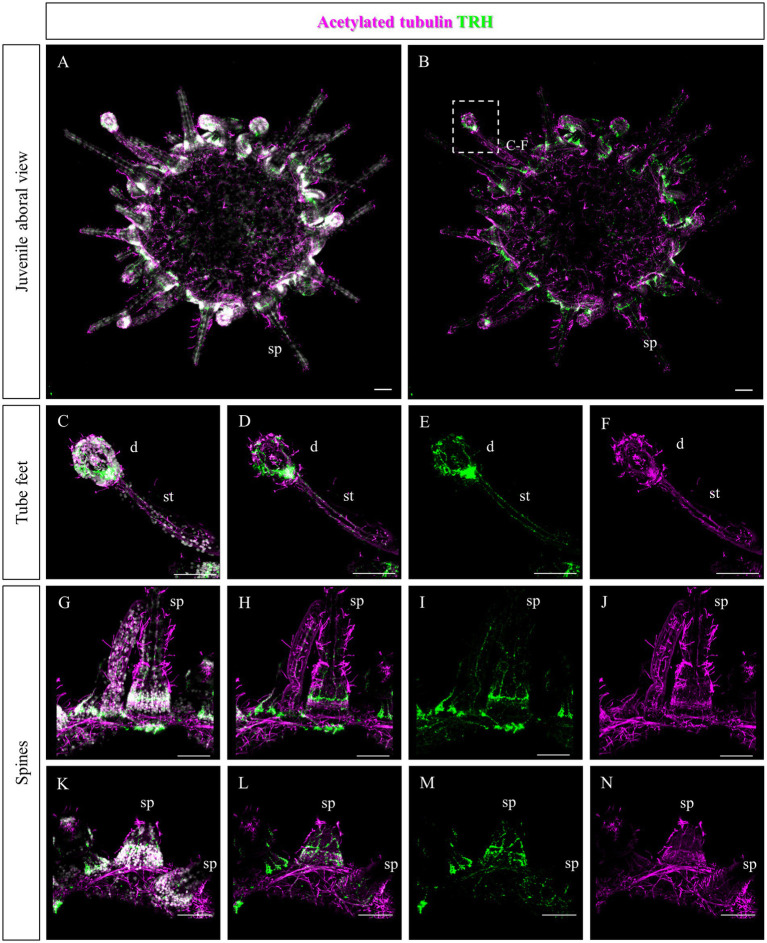
TRHergic cells in *P. lividus* juveniles, aboral view. Double TRH and acetylated tubulin IHC were performed on juveniles. At this stage, acetylated tubulin also stains the nervous system. **(A,B)** Overview of the juvenile from the aboral side. It is possible to see that the aboral epithelium is covered by cilia and some TRH projections are also stained. No spines or tube feet yet cover this side, but it is possible to observe the morphology of the ones located on the sides. **(C–F)** A tube foot is shown form a lateral view. It is possible to appreciate the stalk, in which two parallel nerves positive to TRH run. At the base of the tube foot disk, there is a ganglion of TRHergic cells from which more axons arise to crown the disk. **(G–N)** Spines imaged from the side. A net of TRH positive projections surrounds their surface. Ganglia of TRHergic cells are located below the spines in **(G–J)** and connect to the epithelial network. Nuclei are labeled with DAPI (in white). All pictures are maximum projections of confocal images. sp., spine; d, disk; st, stalk, scale bars = 50 μm.

**Figure 6:**
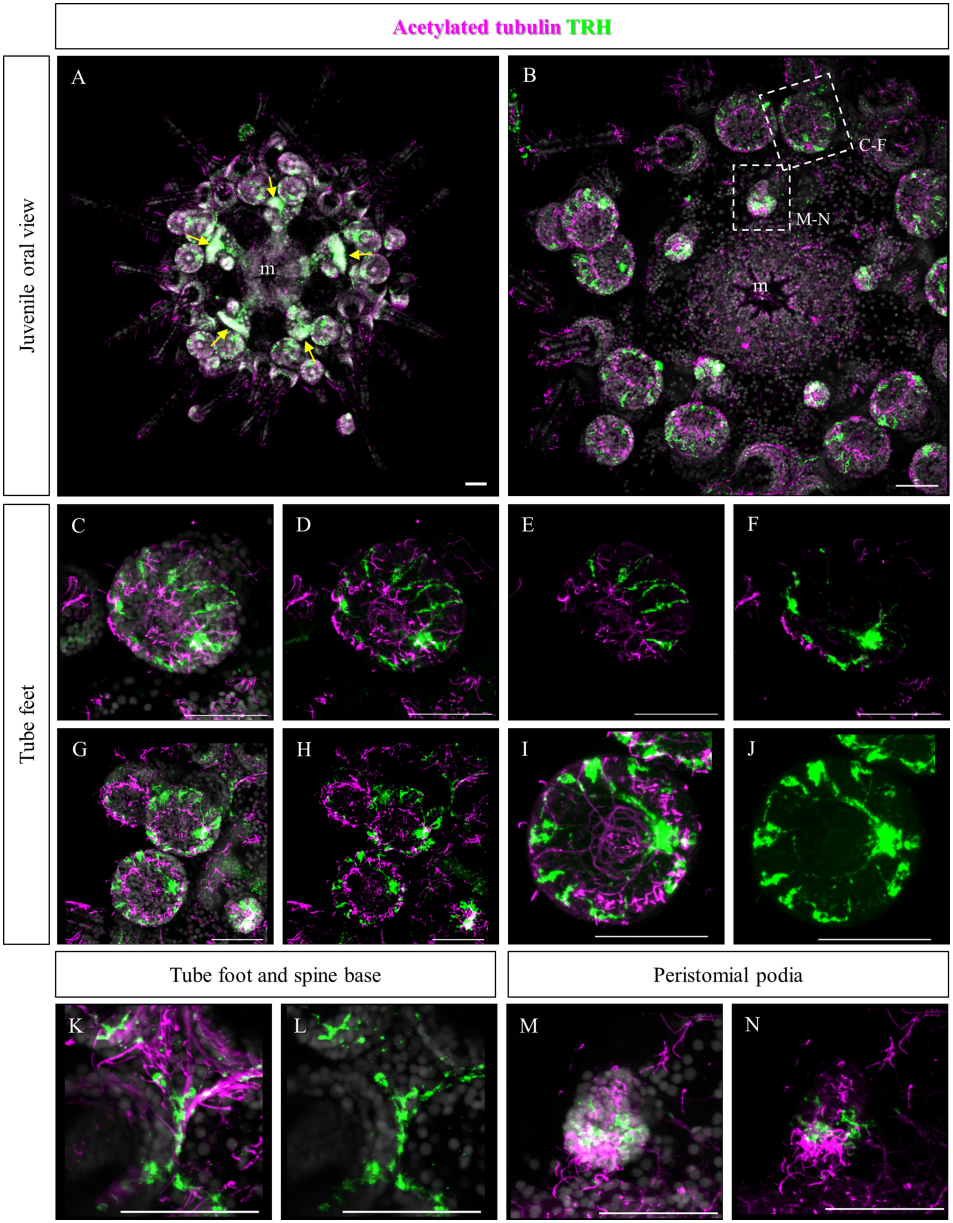
TRHergic cells in *P. lividus* juveniles, oral view. Double TRH and acetylated tubulin IHC were performed on juveniles. **(A)** An overview of the oral side of the juvenile is shown. At the center the mouth is surrounded by five peristomial podia and the five radial nerves are also visible. At the tip of each radial nerve there is a group of three tube feet. Between these a group of four spines emerges. Yellow arrows indicate the radial nerve tips. **(B)** A detail of the oral ectoderm is shown, focusing on the epidermis. **(C,D)** A tube foot is imaged slightly from the side. It is possible to see a group of TRHergic cells and other elongated cells surrounding the crown. **(E,F)** Are partial projections of the tube foot shown in **(C,D)**, to highlight its morphology. Panel **(E)** is focused on the epithelium that covers the side of the disk. It is possible to observe the morphology of the ganglion from which TRHergic axons depart. In **(F)**, it is possible to appreciate the morphology of the ganglion from which TRH positive axons depart. **(G,H)** More tube feet are shown. In the bottom right corner, there is also a buccal tube foot, and just above a radial nerve is visible too. **(I,J)** Detail of the tube foot on the bottom in **(G,H)** imaged from the front of the disk. It illustrates how the TRHergic crown of cells is located on the epithelium, and how they connect to the ganglion at the base of the disk. **(K,L)** TRHergic cells and projections run in the epithelium at the base of tube feet and spines. **(M,N)** A close up of a peristomial podia, showing that it has a ciliated tip. Two groups of TRHergic cells are located underneath this ciliated tip. Nuclei are labeled with DAPI (in white). All pictures are maximum projections of confocal images. m, mouth, scale bar = 50 μm.

**Figure 7:**
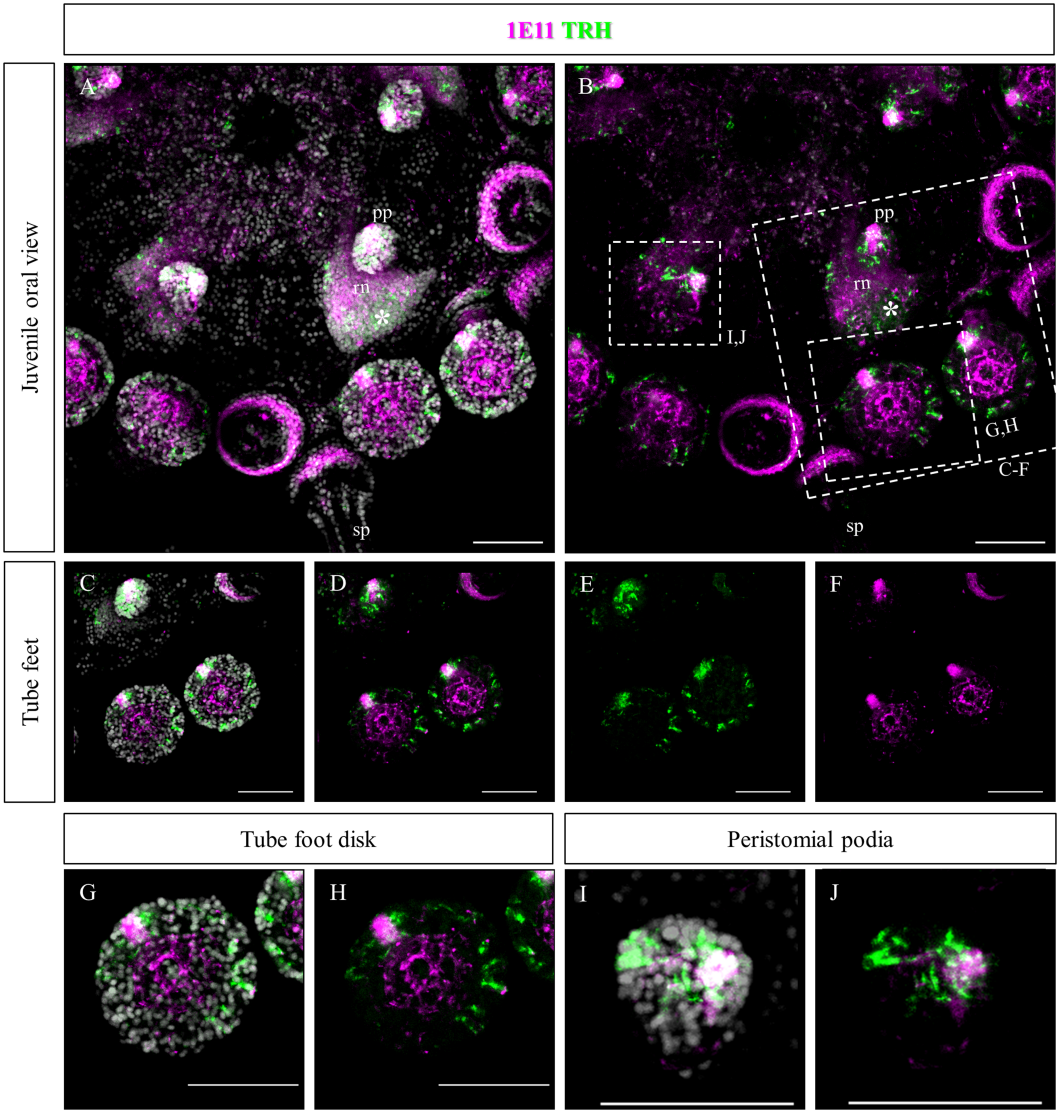
Double staining of TRHergic and SynB in juvenile nervous system of *P. lividus*. Double IHC was performed to stain TRHergic cells in combination with SynB antibody (1E11) as a pan-neuronal marker. **(A,B)** Juvenile seen from the oral side. 1E11 stained the radial nerves, a thick bundle at the base of the spines, the central area of tube feet and a ganglion in the tube foot disks and in the buccal tube feet. **(C–H)** Close ups of tube feet and their disks show that TRHergic cells are located externally to the central disk of fibers. Moreover, not all the cells of the ganglion at the base of the disk are TRHergic, and vice versa. **(I,J)** Detail of a peristomial podium shows that only one of the TRHergic group of cells is also labelled by 1E11. Nuclei are labeled with DAPI (in white). All pictures are maximum projections of confocal images. sp., spine; pp., peristomial podia; rn, radial nerve; scale bar = 50 μm.

Since no TRHergic cells were observed in the rudiments, we set out to investigate if the TRH-type neuropeptide is expressed after metamorphosis in *P. lividus*. Therefore, immunostaining was performed on juveniles a few weeks post metamorphosis, corresponding to stage J5-J6 in Thompson et al. (2021). Sea urchin juveniles at this stage look very much like adult sea urchins on a smaller scale. They have five sets of three tube feet on the oral side, in correspondence with each radial nerve, and five sets of four spines between them. Moreover, they have five peristomial podia located around the mouth, in correspondence with the radial nerves. On the contrary, the aboral surface does not have spines yet at this stage. More spines and tube feet are located on the lateral side of the juvenile. The TRHergic cells appear to be quite widespread. In fact, TRHergic projections were stained on the aboral side of the juvenile (Figures 5A,B). TRHergic axons also innervate the stalk of tube feet (Figures 5C–F). In the tube feet observed from the aboral side, TRH cell bodies can be observed at the base of the disk, and additional projections seem to branch from this, up into the disk (Figures 5C–F). At juvenile stage, the anti-acetylated tubulin stains not only cilia, but also neuronal fibers, therefore the anti-acetylated tubulin antibody was used to highlight juvenile morphology. Another group of TRHergic cells is located underneath the spines, forming a ganglion (Figures 5G–N). Moreover, a net of TRHergic projections extends to the spines (Figures 5G–N). On the oral side, a group of TRHergic neurons are located at the tip of the radial nerve (Figure 6A, see yellow arrows). In addition, two groups of TRH positive cells are located at the tip of the peristomial podia (Figures 6B,M,N). Ganglia containing TRHergic cells are also localized at the base of the tube foot disks (Figures 6C–J). From these ganglia, TRHergic projections branch to the sides of the discs, where cell bodies of elongated bipolar cells are located (Figures 6C–J). Additionally, TRH projections are located at the base of the tube feet (Figures 6K,L). In order to check if the juvenile’s TRHergic cells are neuronal cells, immunostaining against an echinoderm pan neuronal marker (synaptotagmin-B or SynB) was performed using the antibody 1E11 (Burke et al., 2006b). In our juveniles, 1E11 effectively stained the radial nerves, the axons at the base of the spines, and the ganglia located in the peristomial podia, and at the base of the disks in the tube feet, in agreement to what previously described at slightly earlier juvenile stage by Burke et al. (2006a), Formery et al. (2021), Paganos et al. (2022c). Intriguingly, not all the TRHergic cells were found positive to SynB (Figures 7C–H). In particular, the tube feet ganglia showed co-expression only in some cells located around the center. Also, in the peristomial podia, only one of the of the two TRHergic groups of cells looks to be positive to 1E11 (Figures 7I,J). Since 1E11 is commonly considered a pan-neuronal marker, this could suggest that not all the TRHergic cells observed at juvenile stage are neurons or utilizing different synaptotagamins. Indeed, it has been shown by recent studies that SynB is not the only synaptotagamin present in the echinoderm nervous system (Meyer et al., 2023) and that 1E11 is not sufficient to label all the neuros present in the tube foot disk (Paganos et al., 2022c). Nonetheless, further analysis, possibly including scRNA-seq, are necessary to confirm the possibility that non-neuronal population(s) of TRHergic cells also exists.

### 2.3 Molecular characterization of the TRHergic cells at early larva stage in *Paracentrotus lividus*

**Figure 8:**
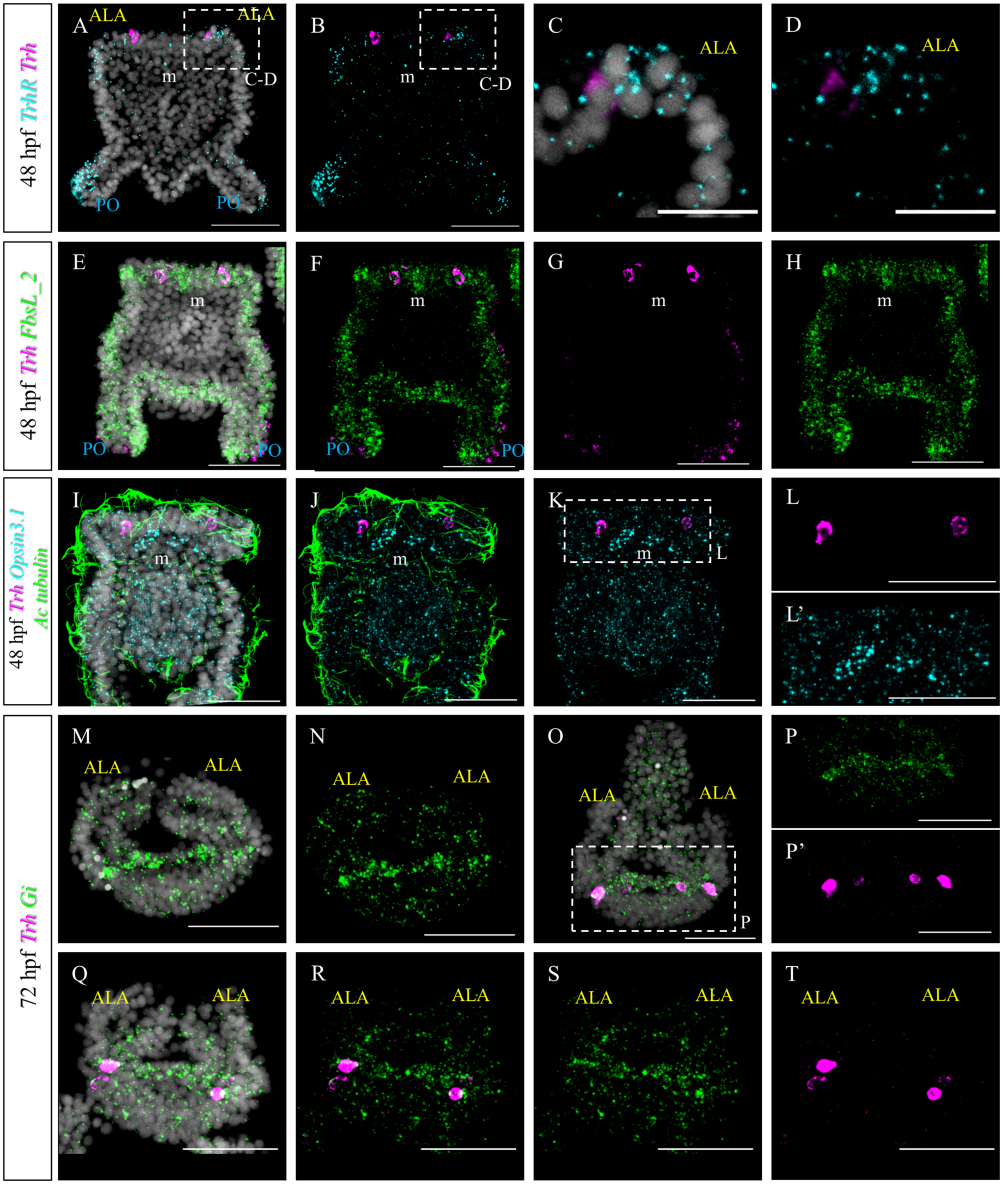
Characterization of the TRHergic cells at the early pluteus stage *P. lividus*, first section. **(A–D)** Double FISH for *Trh* and *Trh receptor* was performed at 48 hpf showing that the *TrhR* is expressed at the tip of ALA and PO. Some receptor transcripts are located close to the *Trh* + cells, but no clear overlap is observed, suggesting absent or very low co-expression. **(E–H)** Double FISH for *Trh* of the ciliary band marker *FbsL_2* was performed on 48 hpf plutei. **(I–L’)** Double FISH for *Trh* and *Opsin3.1* combined with IHC for acetylated tubulin to stain the cilia were performed on 48 hpf plutei. **(M,N)** FISH for *Gi* was performed at 72 hpf. Larva is shown in an oral view, as in Figure 1C, Figures 2O–P’, and Figure 4I, focusing only on the ALA and apical plate. **(O–T)** Double FISH for *Gi* and *Trh* was performed on 72 hpf plutei. As in **(M,N)**, larvae are shown in an oral view. Nuclei are labeled with DAPI (in white). All pictures are maximum projections of confocal images. m, mouth; ALA, anterolateral arm; PO, post oral arm; scale bars = 50 μm in all but **(C,D)** which have a 20 μm scale bar.

Furthermore, we set out to investigate to what extent TRHergic cell molecular identity and signaling known in *S. purpuratus* (Wood, 2020; Paganos et al., 2021; Cocurullo, 2022; Cocurullo et al., 2023) are conserved in *P. lividus*. In *S. purpuratus* larvae, *TrhR* is expressed at the tip of the ALA and PO (Wood, 2020; Cocurullo, 2022). To determine if this expression pattern is conserved in *P. lividus* larvae, FISH was performed at 48 hpf. This staining confirmed that in both species, *TrhR* is expressed by the arm epithelium (Figures 8A–D). Double staining with *Trh* suggests that TRHergic cells do not express *TrhR*, or, if they do, it is at a very low level (Figures 8C,D). This is especially clear when looking at the individual stacks rather than at the full projections (Supplementary Figure S5A). Double FISH of *Trh* and *FbsL_2* (marker for the ciliary band; Paganos et al., 2021), shows no co-expression (Figures 8E–H and Supplementary Figure S5B), similarly to *S. purpuratus* (Paganos et al., 2021).

Next, we aimed at comparing the molecular signature of *P. lividus’* TRHergic cells with the one described for *S. purpuratus* larvae (Cocurullo et al., 2023). In *S. purpuratus*, the TRHergic cells are photoreceptors expressing the Go-Opsin3.2. Despite the fact that we could identify both *Opsin3.1* and *Opsin3.2* in the *P. lividus’* genome (Opsin 3.2 is pliv13652/ PL31706, Opsin 3.1 is Plv13839/PL31732, https://genome-euro.ucsc.edu; for orthology assignment see D’Aniello et al., 2015), no *Opsin3.2* expression was detected at early larva stage by PCR and RT-PCR (data not shown). However, we could detect *Opsin3.1*, which is not expressed in *S. purpuratus* at early pluteus stage. *Opsin3.1* probe gave a diffused signal throughout the larva, but the signal is mainly accumulated in an area between the two ALA. Since data from our previous work (Cocurullo et al., 2023) suggests that *Sp-Opsin3.2* could signal through a Gi-mediated phototransduction cascade, we also performed a FISH to detect the expression pattern of *Pl-Gi*. Intriguingly, although this *Gi* seems to be broadly expressed, transcripts accumulate in the TRHergic cells and in cells located in the dorsal area between the two ALA, where the apical organ is located (Figures 8M,N). Double *Gi/Trh* FISH confirmed expression of Gi in the TRHergic cells at 72 hpf (Figures 8O–T). In summary, whether *P. lividus* TRHergic cells are photoreceptors remains a mystery and further analysis is required.

**Figure 9:**
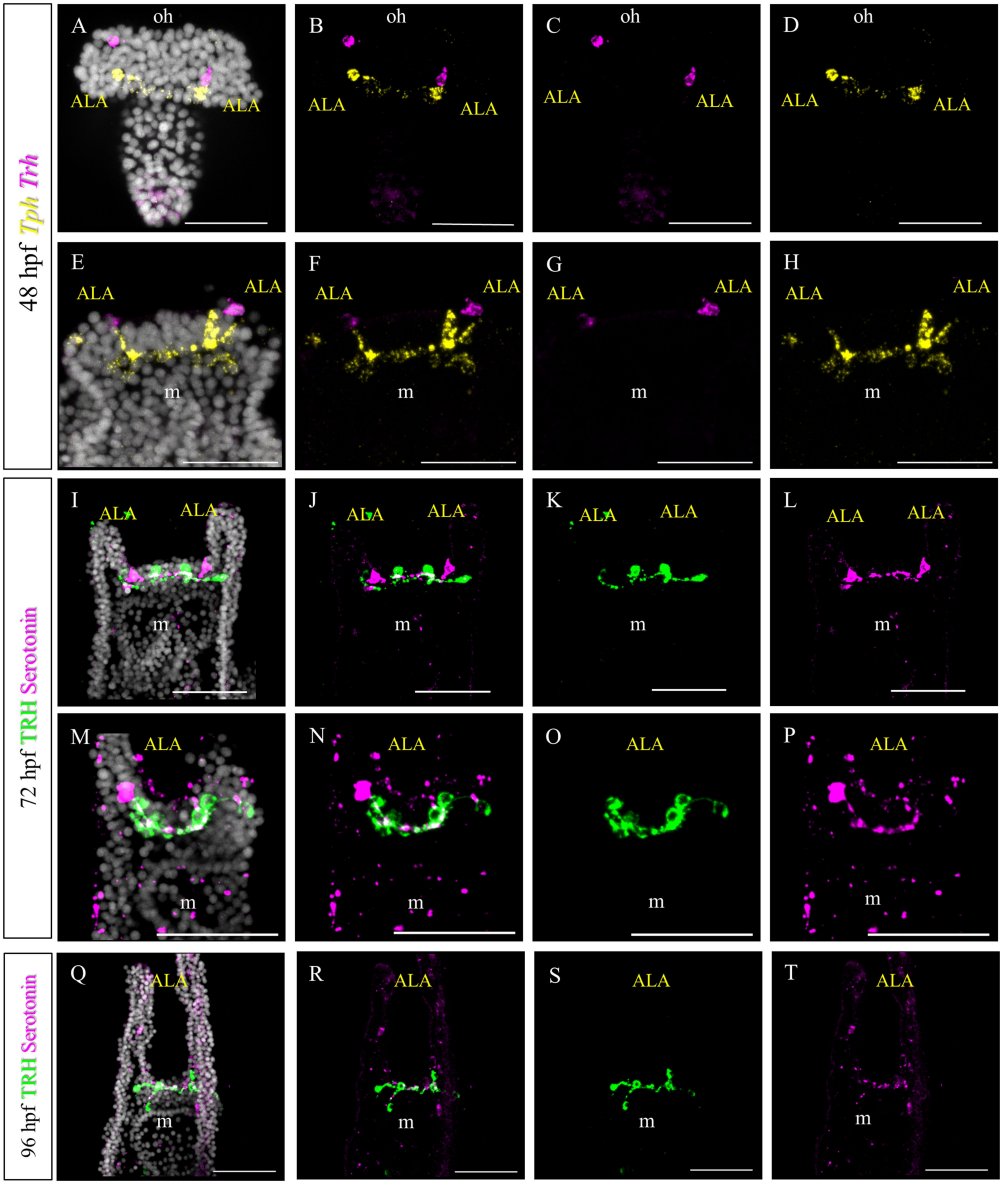
Characterization of the TRHergic cells at the early pluteus stage in *P. lividus*, second section. **(A–H)** Double FISH for *Trh* and *Tph* was performed at 48 hpf. Double IHC for TRH and serotonin was performed on 72 hpf **(I–P)** and 96 hpf **(Q–T)** plutei. Nuclei are labeled with DAPI (in white). All pictures are maximum projections of confocal images. m = mouth, oh = oral hood, ALA = Anterolateral arm, scale bars = 50 μm.

In *S. purpuratus*, TRHergic cells represent a distinct cell cluster, which connects to the apical organ (Paganos et al., 2021). Based on the evidence that in *P. lividus* the first TRHergic cells to appear are located closer to the apical organ than in *S. purpuratus*, we were interested in determining if there is any co-expression of TRH and TPH/Serotonin at early *P. lividus* larva stages. Tryptophan hydroxylase, or TPH, is an enzyme involved in the synthesis of the monoamine neurotransmitter serotonin. At 48 hpf, there are about 2–3 *Tph* huge flask-shaped positive cells located in the apical organ (Figures 9E–H). A number of smaller round shaped *Tph* positive cells are distributed nearby and between the bigger cells. Of these cells, none was also *Trh* positive, although some *Trh +* cells were located in very close proximity to the *Tph +* ones (Figures 9A–D). At 72 hpf, we used an anti-serotonin antibody to identify the serotonergic cells. IHC of Serotonin and TRH at 72 hpf, stained two serotonergic neurons and four TRHergic cells. The TRH positive ones are located two at the base of the ALA and two more externally, below the ALA (Figures 9I–T). The serotonergic cells were located between the more external TRHergic cell and the one located at the arm connection. TRHergic and serotonergic axons in the apical organ seem to run very close to each other. At 96 hpf, often one or more serotonergic cell bodies are stained deep in the ALA (Figures 9M–P). These findings suggest that the TRHergic cells do not belong to the serotonergic cell type in *P. lividus*, similarly to what found in *S. purpuratus* (Paganos et al., 2021; Cocurullo et al., 2023).

**Figure 10:**
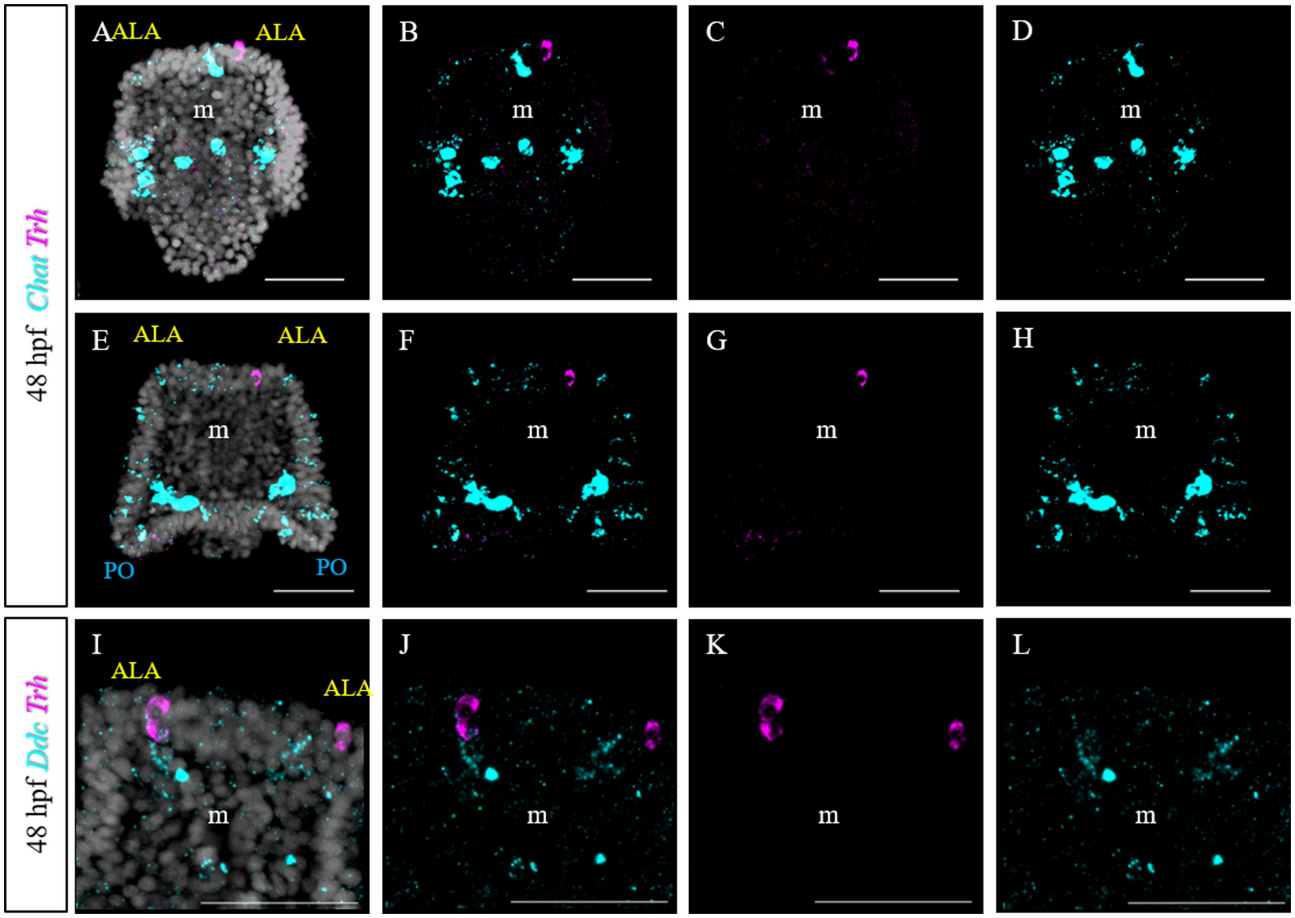
Characterization of the TRHergic cells at the early pluteus stage in *P. lividus*, section 3. **(A–H)** Double FISH for *Trh* and *Chat* was performed at 48 hpf. **(I–L)** Double FISH for *Trh* and *Ddc* were performed on 48 hpf plutei. Nuclei are labeled with DAPI (in white). All pictures are maximum projections of confocal pictures. m, mouth; ALA, anterolateral arm; PO, post oral arm; scale bars = 50 μm.

Furthermore, we tested if *Trh +* neurons at early pluteus stage are also cholinergic, like in *S. purpuratus* (Paganos et al., 2021). To do this, we performed a double FISH for *Trh* and *Chat.* Choline acetyltransferase (commonly abbreviated as ChAT) is a key enzyme responsible for the synthesis of the neurotransmitter acetylcholine and therefore its expression is used as marker to identify cholinergic neurons. In *P. lividus*, at early 48 hpf, no *Trh/Chat* co-expression was detected (Figures 10A–H). Similarly, we tested if TRHergic neurons are dopaminergic using the expression of DOPA decarboxylase (DDC) as marker.

### 2.4 Identification of TRHergic cells in other sea urchin species

**Figure 11:**
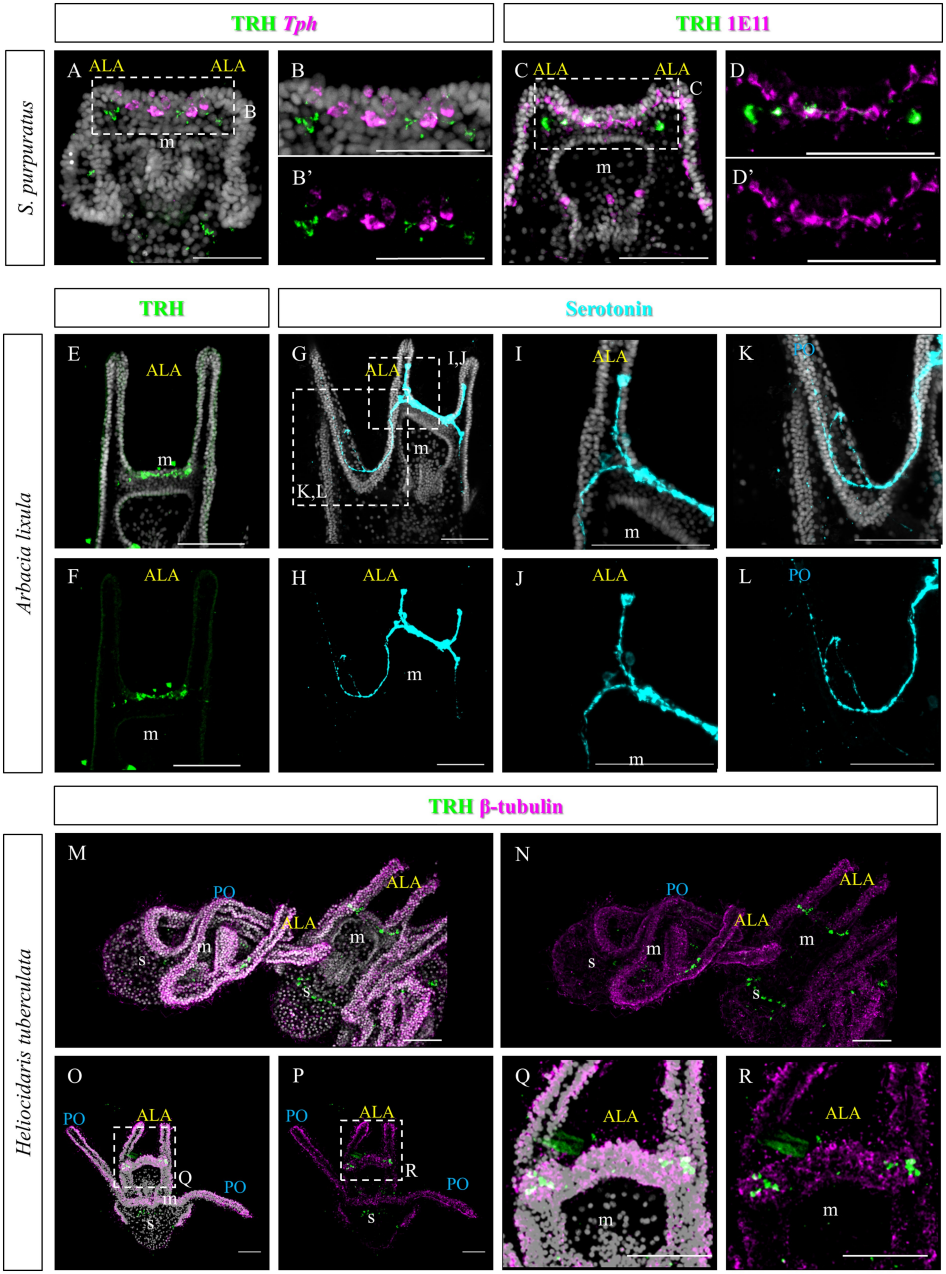
Characterization of the TRHergic cells at early pluteus stage in *S. purpuratus*, *A. lixula* and *H. tuberculata*. **(A,B)** TRH IHC was combined with *Tph* FISH on *S. purpuratus* larvae at 96 hpf. **(C–D′)** Double ICH for TRH and the pan-neuronal marker 1E11 was performed on *S. purpuratus* larvae at 6 dpf. TRH **(E,F)** and Serotonin **(G–L)** immunostaining were performed on *A. lixula* at pluteus stage. **(M–R)** Double IHC for TRH and β-tubulin were performed on *H. tuberculata* at 72 hpf pluteus stage. In **(M)**, two plutei and part of a third in the bottom right, are shown. Nuclei are labeled with DAPI (in white). All pictures are maximum projections of confocal images. m, mouth; s, stomach; ALA, anterolateral arm; PO, post oral arm, scale bars = 50 μm.

As previously described, TRHergic cells in *S. purpuratus* are located at the sides of the serotonergic neurons, at least up to 96 hpf (Figures 11A–B’). Moreover, the number of TRHergic cells appears to increase at a slow rate and at 6 dpf larvae having still two TRHergic neurons can be observed (Figures 11C–D’). Based on these differences observed between TRHergic cells in *S. purpuratus* and *P. lividus*, we set out to investigate the localization of TRHergic cells in two other species.

First we chose *Arbacia lixula*, which is another Mediterranean sea urchin species sharing the same habitat of *P. lividus*. TRH IHC stained a group of projections and cell bodies located between the ALA, resembling TRH expression in *P. lividus* after 1 week post fertilization (Figures 11E,F). Serotonergic cells, on the contrary, show a different organization. Most cell bodies could be stained only at the sides of the area between the ALA (Figures 11G–L). Another serotonergic cell is usually located inside the ALA, next to the tip, that sends a projection toward the cells at the base of the arms. Moreover, from the basal group of cells, axons run into the oral hood and toward the PO arms.

The second species we investigated is *Heliocidaris tuberculata*, a species of the Pacific Ocean, like *S. purpuratus*, but since it comes from Australia, larvae from this species grow at a higher temperature, more similar to the Mediterranean species. In comparison, *Heliocidaris* plutei have long arms, like *P. lividus*, but a more compact and large body, similar to *S. purpuratus*. In *H. tuberculata,* our TRH antibody stained about two cells located at each side of the apical plate (Figures 11M–R), which resemble the topology of the TRHergic cells in *S. purpuratus* plutei.

## 3 Discussion

Sea urchins are useful and interesting organisms to investigate the anatomy of nervous systems, especially from an evolutionary perspective. Moreover, since most sea urchin species are indirect developers, and the larval and adult nervous system are independent and have two completely different modes of organization, they also represent a valuable system to investigate the transition from larval to adult nervous system. Also, the diversity observed among sea urchin species makes them a powerful tool to investigate how evolution works on a smaller evolutionary scale, while their phylogenetic position as deuterostome invertebrates, enables analysis of neural evolution over longer evolutionary timescales. Here we took advantage of the fast development of *P. lividus* to investigate how a specific neuronal TRHergic group of cells progresses across development, from early larva to post metamorphic juveniles. Moreover, focusing on the early larva, we compared the molecular signature of the TRHergic cells in *P. lividus* with that previously described in *S. purpuratus* (Cocurullo et al., 2023), and, to investigate how this cell type evolved, we compared the distribution of TRHergic cells obtained in these two species with that identified by immunostaining in two other sea urchin species, *A. lixula* and *H. tuberculata*.

### 3.1 Development of TRHergic cells from larva to juvenile

Similarly to *S. purpuratus* (Wood et al., 2018; Cocurullo et al., 2023), TRHergic cells in *P. lividus* first appear at early pluteus stage as one or two cells bilaterally located above the mouth (Figure 2). However, while in *S. purpuratus* these first two cells are located in the oral ectoderm just above the mouth, in *P. lividus* they are more dorsal and located right at the corner of the ALA. Very quickly and in a non-stereotypic way, the number of TRHergic cells increases and most larvae at 72 hpf already possess four of them. From this point on, also the net of connections between the cells increases and at late 6-armed larval stage they colonize the oral hood (Figures 3, 4). At this stage, projections from the oral hood extend underneath the ciliary band of all six arms and of the buds of the additional two ALA forming in front of the primary ones. No TRHergic neurons or axons were stained in the rudiment, but juveniles showed an extended network of TRHergic cells and projections (Figures 5–7), raising questions on when the first TRHergic cells appears in the rudiment/juvenile. To answer this question, a more detailed analysis of larvae at 8-armed stage and juveniles during and right after metamorphosis is needed.

### 3.2 TRHergic cells across sea urchins: clues from molecular characterization

Based on the differences observed in terms of topology and number of TRHergic cells at comparable developmental stages in *S. purpuratus* and *P. lividus*, we decided to further investigate the molecular identity of the TRHergic cells in *P. lividus*.

First we investigated the expression pattern of the TRHR. Similarly to what was observed for *S. purpuratus* (Cocurullo, 2022), the receptor at early larva stages is expressed on the epithelium which covers the tip of ALA and PO, suggesting that the function of the TRH neuropeptide is conserved between *P. lividus* and *S. purpuratus* (Figure 8). Here, we also set out to compare the molecular fingerprint of the TRHergic cells of *P. lividus* and *S. purpuratus*. Single cell transcriptomics of *S. purpuratus* larvae at early pluteus stage (72 hpf) identified the TRHergic cells as an independent neuronal subcluster (called distal apical plate neurons) (Paganos et al., 2021). In fact, these cells, despite their proximity to the ciliary band and the serotonergic neurons of the apical organ, did not express genes such as *Sp-FbsL_2* and *Sp-Tph*, which are markers for the ciliary band and serotonergic neuron clusters, respectively. Intriguingly, also *P. lividus* plutei at 48 hpf (which is the corresponding early pluteus stage) do not co-expressed *FbsL_2* or *Tph* (Figures 8, 9), suggesting that the TRHergic cells could form an independent neuronal cluster in *P. lividus* too. Moreover, the relative position of TRHergic and serotonergic neurons seems to be slightly different between the two species. While in *S. purpuratus*, serotonergic neurons are located in between the two lateral clusters of TRHergic cells (Figures 11A,B; Wood et al., 2018), in *P. lividus*, the serotonergic cells look to be located more at the sides of the oral hood, and from later stages on (72–96 hpf), some cells are also localized in the ALA (Figure 9). Nonetheless, up to 96 hpf, TRH and *Tph*/Serotonin do not co-express in the same cells (Figures 11A,B), although their axons and cell bodies appear to be in very close proximity in the central area between the ALA. Furthermore, we investigated if the TRHergic cells in *P. lividus* are also secreting neurotransmitters, in particular acetylcholine and dopamine. For this, we performed a double FISH of *Trh* and *Chat* (key enzyme for acetylcholine synthesis) and *Ddc* (enzyme for dopamine synthesis). In both cases, no co-expression with TRH was observed, although some dopaminergic neurons appear to be located in very close proximity to the TRHergic cells (Figure 10).

An interesting characteristic of the TRHergic neurons of the *S. purpuratus* larva is their dual sensory/neurosecretory role as they also express the Go-Opsin3.2 and deploy an ancient regulatory module responsible for photoreceptor specification (Cocurullo et al., 2023). Therefore, we investigated if the TRHergic cells in *P. lividus* are also photoreceptors. Interestingly, it was impossible for us to detect the expression pattern for the *Opsin3.2*, both by immunostaining or FISH (data not shown). Nonetheless, *S. purpuratus* genome encodes a second Go-Opsin named Opsin3.1, which is not expressed at early pluteus stage. Also *P. lividus* genome encodes an orthologous *Opsin3.1*. Our data suggest that *Opsin3.1* is expressed at the pluteus stage, but the expression pattern is different from the *Sp-Opsin3.2* (Figure 8). In fact, *Opsin3.1* transcripts are found in a few cells located between the two ALA in *P. lividus*, while the co-expression with *Trh* is not yet clear. Indeed, single-cell transcriptomic is a powerful technique that will allow us to answer this question in the near future. Nonetheless, this finding shows a major difference between *S. purpuratus* and *P. lividus* larvae since it means that these two species deploy a different Go-Opsin at the pluteus stage, and most likely with different functions. Also, the expression pattern of these Go-Opsins is different, suggesting a fascinating evolutionary history for Go-Opsins in sea urchins. We could hypothesize that after gene duplication, Go-Opsins were differentially deployed. Nonetheless, the *Opsin3.*1 expression profile in *P. lividus* represents the first evidence for this process, since a Go-Opsin of another sea urchin species (*Hemicentrotus pulcherrimus*), closely related to *S. purpuratus,* appears to have the same expression pattern as *Opsin3.2* in *S. purpuratus* (Yaguchi and Yaguchi, 2021).

Nonetheless, TRHergic cells in *P. lividus* could be sensory as they are ciliated. To further investigate if these cells could be photoreceptors, we also tested the presence in these cells of transcripts derived from homologues of retinal genes found to be expressed by the Sp-Go-Opsin3.2+ cells of 72 hpf and 5 dpf *S. purpuratus* larvae (Paganos et al., 2021; Cocurullo et al., 2023). In *S. purpuratus*, the phototransduction cascade activated by Opsin3.2 might be mediated by the Gi protein; therefore, we investigated the expression of *Gi* in the 4-armed *P. lividus* larva. Intriguingly, in these larvae, *Gi* transcripts are accumulated in cells located between the ALA, in a similar position to *Opsin3.1* and *Tph* positive cells, but also in the TRHergic cells (Figure 8).

**Figure 12:**
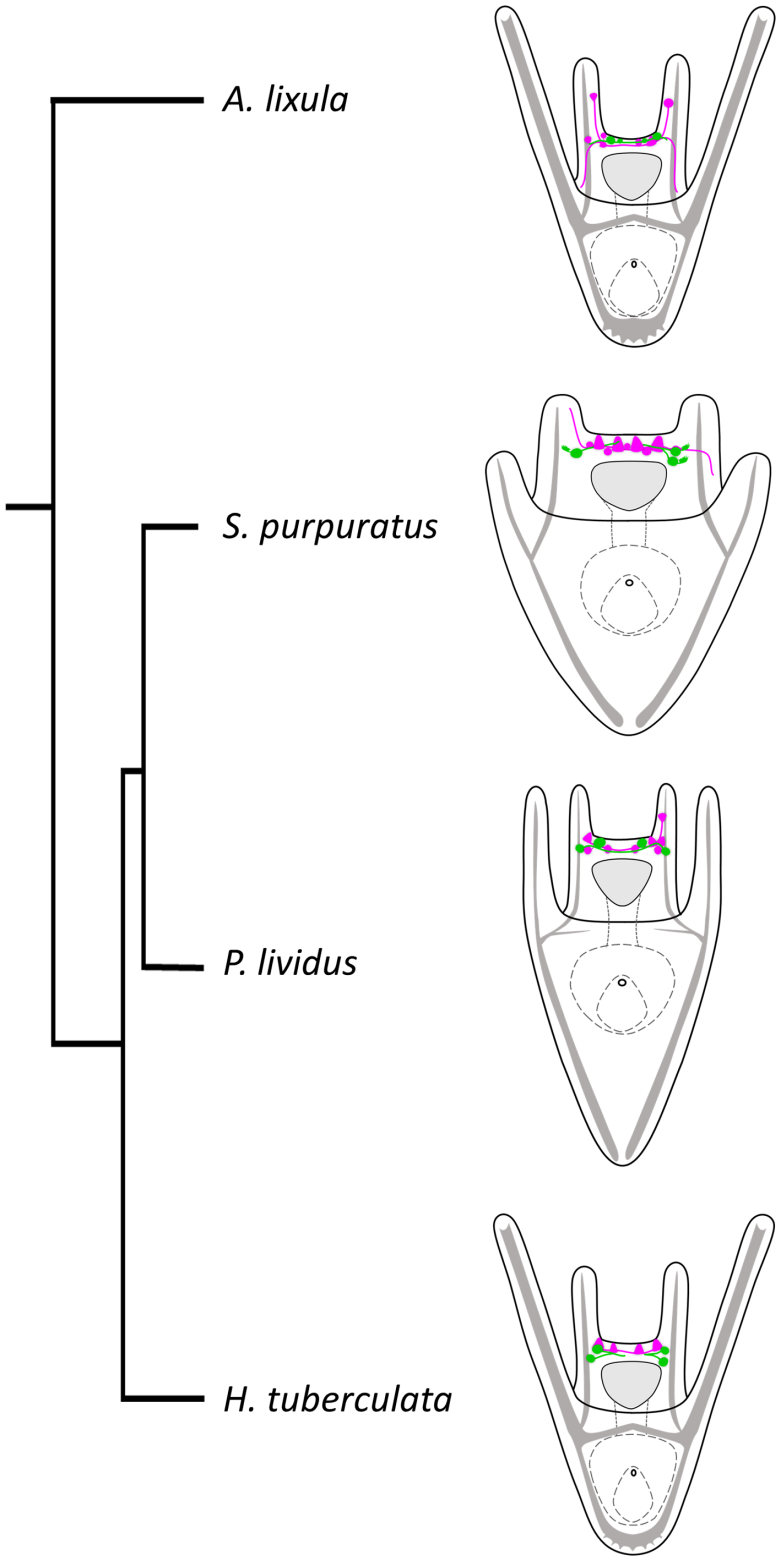
Organization of TRHergic and serotonergic neurons across sea urchin species. Distribution of serotonergic cells (shown in magenta) in relations to TRHergic cells (in green) in the different sea urchin species, based on findings of this study or deduced from previous studies (Bisgrove and Raff, 1989; Byrne et al., 2007; Rendell-Bhatti et al., 2021; Tsironis et al., 2021). The phylogenetic relationships between the four sea urchin species were deduced from Littlewood and Smith (1995) and Ziegenhorn (2017).

Finally, we performed a survey to assess the localization of TRHergic cells also in other sea urchin species, namely *A. lixula*, *H. tuberculata,* and compared their topology with that observed in *P. lividus* and *S. purpuratus*. We also attempted to reconstruct the relative localization in relation to the serotonergic neurons in these different larvae. We summarized these results in the illustration shown in Figure 12, with the integration of a phylogenetic tree showing evolutionary relations of these four sea urchin species. It is important to note that data regarding serotonergic neuron organization in *A. lixula*, *H. tuberculata,* and *P. lividus* are extremely limited or absent, and need to be expanded (Bisgrove and Raff, 1989; Byrne et al., 2007; Rendell-Bhatti et al., 2021; Tsironis et al., 2021). Nonetheless, our comparison suggests that the relative organization of TRHergic and serotonergic cells slightly changes in different sea urchin species, being the two types of neurons more intermingled in *P. lividus* and *A. lixula* as compared to the more clustered distribution of these cells in *S. purpuratus* and *H. tuberculata* at the early larva stage. The mechanistic meaning of such subtle differences need to be further investigated. On the other hand, the comparison of different species would provide new evidence to understand how the TRHergic/photoreceptor cells evolved.

## 4 Conclusion

In this work, we could follow the progression of TRHergic cells during the development of the invertebrate deuterostome *P. lividus* from larva to juvenile stages, thus providing a first in depth characterization of these cells across different life stages in a sea urchin. Moreover, comparing the topology and molecular identity of the TRHergic neurons in *P. lividus* with what is observed in other sea urchin species, and in particular with that of the Sp-GoOpsin3.2/TRHergic cells, we highlight the value of these animals to study how nervous system and sensory/neurosecretory cells evolve over a relatively short evolutionary timescale.

## 5 Materials and methods

### 5.1 Animal husbandry

*Paracentrotus lividus* gametes were obtained by the Stazione Zoologica Anton Dohrn animal models facility. Spawning was induced by intracoelomic injection of 0.5 M KCl. The sperm was collected dry using a Pasteur pipette and stored at 4°C until usage. To collect eggs, females were inverted over a beaker filled with Mediterranean Sea seawater (FMSW). About 20 mL of eggs were fertilized, adding a few drops of sperm diluted 1:10000. Embryos were transferred in FMSW and reared at 19°C under a 12 h light/12 h dark cycle. To grow larvae until metamorphosis, specimens were reared in 2 or 4 L glass beakers and fed three times per week with a mix of 70% of *D. tertiolecta* and 30% *Isochrysis galbana*. The amount of algae was increased from 5,000 cells/ml to 80,000–10,000 cells/ml, according to the needs of the cultures as described in Zupo et al. (2018). Cultures were cleaned two times per week removing most of the sea water and replacing it with fresh MFSW. After metamorphosis, juveniles were fed with seaweed.

Adult *S. purpuratus* were obtained from Patrick Leahy (Kerckhoff Marine Laboratory, California Institute of Technology, Pasadena, CA, USA) and maintained in circulating seawater at Stazione Zoologica Anton Dohrn in Naples. Gamete release was induced by vigorously shaking the adults. Gametecollection, fertilization and culture maintenance were performed as described for *P. lividus* but using Mediterranean Sea water diluted 9:1 to adapt to the different salinity of the Pacific Ocean.

*A. lixula* gametes were obtained as for *P. lividus* and the larvae reared in the same conditions, however, they were not maintained to metamorphosis, but they were collected at 4-armed pluteus stage.

*Heliocidaris erytrogramma* samples, fixed at 72 hpf for *in situ* were kindly gifted by Prof Maria Byrne, University of Sydney.

### 5.2 Fluorescent *in situ* hybridization and immunohistochemistry

Specimens for immunostaining (IHC) were fixed in 4% PFA made in MFSW at Room Temperature (RT). The incubation time was adapted to the size of the specimens. For pluteus stages, 10–15 min were used, while at later pluteus stages the incubation time was increased up to a 20 min. After the incubation, the samples were thoroughly washed with PBS and stored at 4°C. Juveniles were fixed for 15 min in 4% PFA, washed once with PBS to remove the fixative and then incubated in cold methanol for 15 min on ice. Finally, they were washed 3–4 times with PBS and stored at 4°C. Specimens for *in situ* hybridization were fixed in Fixative I (4% PFA in 0.1 M MOPS and 0.5 M NaCl) for at least one night at 4°C. Subsequently, samples were washed 3 times with MOPS buffer (0.1 M MOPS, 0.5 M NaCl, 0.01% Tween 20) for 15 min at RT, dehydrated in 70% ethanol and finally stored at-20°C until usage.

Whole mount RNA Fluorescent *in situ* hybridization (FISH) and combined FISH-IHC were performed as described in details in Perillo et al. (2021) and Paganos et al. (2022a). Summarizing, antisense probes were transcribed from linearized DNA and labelled during transcription using Digoxigenin (Roche) or Fluorescein (Roche) labelled ribonucleotides following the manufacturer’s instructions. *Pl-Trh* sequence was obtained by blasting the *Sp-Trh* sequence against the Trinity transcriptome, the full sequence is included in the Supplementary materials (TRH CDS sequence in *P. lividus*) together with the translated protein sequence (TRH peptide sequence in *P. lividus*) and the TRH precursor protein sequences from *P. lividus* and *S. purpuratus* aligned and annotated (Aligned TRH precursor peptide sequences in *P. lividus* and *S. puprupartus*). Primer sequences used for cDNA isolation and probes synthesis and the type of ribonucleotides used are included in Supplementary Table S1. Fluorescent signal was developed via using fluorophore conjugated tyramide technology (Perkin Elmer, Cat. #NEL752001KT). For combined FISH-IHC, after tyramide amplification step, samples were incubated in blocking (containing 1 mg/ml Bovine Serum Albumin and 4% Sheep Serum in PBS) for 1 h at RT, then transferred in primary antibody diluted 1:400 in blocking O/N at 4°C. The list and details of the primary antibodies used can be found in the Supplementary Table S2 Samples were washed 4–6 times with PBS 1x, then stained with appropriate Alexa Fluor secondary antibodies diluted 1:1000 in blocking, and finally washed 4–6 times with PBS 1x. DAPI (10 mg/mL stock) was added to the samples at a final dilution of 1:10000 to stain nuclei. Specimens were imaged using a Zeiss LSM 700 confocal microscope and pictures analyzed using ImageJ 1.53v.

### 5.3 Scanning electron microscopy and whole animal freeze-fracture

*Paracentrotus lividus* larvae at 72 hpf were collected and processed as described in Paganos et al. (2023). In summary, whole plutei were fixed in 2.5% glutaraldehyde in MFSW at 4°C overnight. The day after, samples were washed several times with MFSW and infiltrated with DMSO by subsequent incubations in 25% DMSO in double distilled water (ddH_2_0) for 1 h at RT followed by a second incubation in 50% DMSO in ddH_2_0 for 1 h at RT. During the last 30 min of incubation a metal key, a pair of forceps, an aluminum tray and a razor blade were placed in a polyurethane ice bucket and covered with liquid nitrogen to pre-cool. Approximately 100 μl of specimens were quickly transferred inside the groove of a pre-chilled metal key and spread throughout the groove surface. The key was quickly placed inside a pre-chilled aluminum tray and, using a hammer, a razor blade placed on the key’s groove was stricken multiple times until complete fracturing of the pellet. The fractured sample was then collected and transferred in a clean Eppendorf tube. DMSO solution was removed, and specimens were dehydrated in a graded ethanol series. Samples were then subjected to the standard processing for SEM including critical point drying and sputtering and were analyzed using a Jeol field emission scanning electron microscope (JSM 6700-F).

## Data availability statement

The original contributions presented in the study are included in the article/Supplementary material, further inquiries can be directed to the corresponding author.

## Author contributions

MC: Conceptualization, Formal analysis, Investigation, Methodology, Writing – original draft, Writing – review & editing. PP: Data curation, Formal analysis, Methodology, Writing – review & editing. GB: Data curation, Methodology, Writing – review & editing. MIA: Conceptualization, Funding acquisition, Supervision, Writing – review & editing.
