# Supplementary material for: Characterization of thyrotropin-releasing hormone producing neurons in sea urchin, from larva to juvenile
Source: Front Neurosci. 2024 Apr 10;18:1378520. doi: 10.3389/fnins.2024.1378520 (PMC11039832; doi:10.3389/fnins.2024.1378520)
Supplement: Supplementary file 1 [file Data_Sheet_1.DOCX]

Characterization of Thyrotropin-Releasing Hormone (TRH) producing neurons in sea urchin, from larva to juvenile

Supplementary Material

**Maria Cocurullo^1^, Periklis Paganos^1^, Giovanna Benvenuto^1^ and Maria Ina Arnone^1^**

^1^Department of Biology and Evolution of Marine Organisms, Stazione Zoologica Anton Dohrn, Villa Comunale, 80121, Naples, Italy; [maria.cocurullo@szn.it](mailto:maria.cocurullo@szn.it), [periklis.paganos@szn.it](mailto:periklis.paganos@szn.it), [giovanna.benvenuto@szn.it](mailto:giovanna.benvenuto@szn.it), [miarnone@szn.it](mailto:miarnone@szn.it).

*Correspondence: [miarnone@szn.it](mailto:mia.arnone@szn.it)

**TRH CDS sequence in *P. lividus*:**

>P_lividus_TRINITY_DN699574_c1_g1_i2

TCGTACAGGTGTTAGAACGTGGACAGAGGAGAAAGGGGGAAACAAGGGAAAAACAAACGAGGAAAAAAGGCAAAAAAGAAAAGGCGATCAAACTGAAAGTGGAACCCGAAACTCATCCGCTGGTGGACTCGTTAGTTTTGAAGGTTGAAACTCACTTTACTTGCTCGTCAGTTGACAGACAAACCTAGATGAGCGATTAGGAGGACTAACGCAAATCTTTACGTGTGGGACTTAGCAAGAGGGCGTGGCCATGAAGGGGACAATGGTATTTTATCTGTGGGCTTGTATTCTGGGATACATCACATGGAGTGGGACCGCACTACCGACCGTCCTGGGAAAGGAACTCGTAATTACAGAGGATGATGAACCAGATATAGCTGGCTGGGACCAGGGAGCGGAGATCCCTCTCAGGCATCATTTTTGGGGTGACATAGCAGACGCAGCCGAGGAAGAAGAACTTGACATGTTGTCCCCTGACTCCGACAAACGTCAGTACCCAGGTGGTAAAAGACAGTATCCAGGAGGCAAGCGCCAATACCCTGGGGGCAAACGACAATACCCCGGCGGTAAGCGTCAGTACCCCGGTGGCAAAAGGCAATTCGTCGGAGGAGAACTTATACCTTCCCCCGAGTTACGTCAATACCCCGGTGGAAAGCGTCAATACCCCGGTGGCAAGCGTCAGTACCCCGGCGGCAAGCGTCAATATCCAGGTGGTAAAAGGCAATATCCCGGCGGCAAAAGGCAATACCCTGGTGGCAAACGTTCGGAGGGTAACCAAGATCTCCTGCCTATGGAAATCCGGCAATACCCTGGTGGTAAACGTTCGGAGGGTGACCAAGACCTCCTGCCTATGGAAATCCGGCAATACCCTGGTGGCAAACGTTCAGAGGGCGACCAAGACCTCCTACCTATGGATGGTGGTAAACGTCAGTACCCTGGTGGCAAACGACAATGGCCTGGTGGAAAGAGGCAGTACCCTGGAGGAAAGCGACAGTGGCCCGGCGGAAAGCGACAATTTCCTGGCGGCAAACGCCAATTCGTCGGCGGGGAAGCGTTCGAACAGGAAGCGAACATCAACAAGCGATTCGTCCCTGAGGACGAGACCATGGACTTTTTAAGGCTATCCCAGCTTTATGACACGAACGAAAATCTAGCCGCAGATGAAGAAGAATTAGCCATTGAGGATCTGCTGGATGACATCATGGTTGACACAAGACCAGAAATGGAAGAACCCATGGACATGTTACTAGGCGATGTTGATTCAGAAGATGCTCTTGCGTTGGATTTATCTGCCTTACTTGGCAACAGAACCCCGATCAATGACTGGTAACACTCATCATGGGTCATTCTCTGCTTCATTGTCATTCTTTTAACAGAATATTTTCGAAAAAGATATCATGAGAATAAAAAATTATTACACTTTACCAAGGATCGTTTACAGGTTAGCTGTTCTCAAACAAATTTAGTTCATCCTCGTCATGCCTTTTCTGAAATTGTGTTTGAAACTAAATACAATTTCAATACGTTTGGTTTGTTCTAAACATTTTGTTCCTGGTTATTTTCCGCTTAGTTAGAGTAGGTATATTTATCAAAAGCTTGAGGGAGGCAAAAGAGTGTCCAGAAAGAGAGACAATATTCATTAGCACCTTTTAATTCAACCATACGACTCCTAGATTAAATGAGTGTAAAAACCTAACATAGAAAGAATCCATTCAACACCTATGCTCCAACGTATGTTAGTGTCAATCGACAACTCAATGGCTATTGGTATAAGACATGTATGGAAATGAAAGAAAATGCTCCATTTAGATTTGAATTTATTTTCTTCTTATCTCGATAAAGATATATATTTGTAAATATATATAGAAGGCACTGTTTATAATTATATTATGTTAGTAACTTGTGTAAAGATACTTGTAAGAGTTTATACTGTGTTTGTCTCTTGGACGATCTCGTTTGCACTCGTAAGGAATAAACTGAGATGATTTTAAAAGACCACATTTCTGCCCTGTAAGAACTGGAAATCCGTCCTGTCATTCTAATTGTTTCATGTTCTGTCGATAAAAAAAAGTGTTATTAGTTGTCAACCATTTAGTGATTCTAAAAGTCTCAATCTAATTCGTGCGTTTCATAATACATGCGCCACCAACGTTCGTCATCATGAGAGGTCTATTTGGTCGACAACT

**TRH precursor peptide sequence in *P. lividus*:** translated using Expasy (<https://web.expasy.org/translate/>). In red it is highlighted the actual putative TRH precursor sequence.

R T G V R T W T E E K G G N K G K T N E E K R Q K R K G D Q T E S G T R N S S A G G L V S F E G **Stop** N S L Y L L V S **Stop** Q T N L D E R L G G L T Q I F T C G T **Stop** Q E G V A Met K G T Met V F Y L W A C I L G Y I T W S G T A L P T V L G K E L V I T E D D E P D I A G W D Q G A E I P L R H H F W G D I A D A A E E E E L D Met L S P D S D K R Q Y P G G K R Q Y P G G K R Q Y P G G K R Q Y P G G K R Q Y P G G K R Q F V G G E L I P S P E L R Q Y P G G K R Q Y P G G K R Q Y P G G K R Q Y P G G K R Q Y P G G K R Q Y P G G K R S E G N Q D L L P Met E I R Q Y P G G K R S E G D Q D L L P Met E I R Q Y P G G K R S E G D Q D L L P Met D G G K R Q Y P G G K R Q W P G G K R Q Y P G G K R Q W P G G K R Q F P G G K R Q F V G G E A F E Q E A N I N K R F V P E D E T Met D F L R L S Q L Y D T N E N L A A D E E E L A I E D L L D D I Met V D T R P E Met E E P Met D Met L L G D V D S E D A L A L D L S A L L G N R T P I N D W **Stop** H S S W V I L C F I V I L L T E Y F R K R Y H E N K K L L H F T K D R L Q V S C S Q T N L V H P R H A F S E I V F E T K Y N F N T F G L F **Stop** T F C S W L F S A **Stop** L E **Stop** V Y L S K A **Stop** G R Q K S V Q K E R Q Y S L A P F N S T I R L L D **Stop** Met S V K T **Stop** H R K N P F N T Y A P T Y V S V N R Q L N G Y W Y K T C Met E Met K E N A P F R F E F I F F L S R **Stop** R Y I F V N I Y R R H C L **Stop** L Y Y V S N L C K D T C K S L Y C V C L L D D L V C T R K E **Stop** T E Met I L K D H I S A L **Stop** E L E I R P V I L I V S C S V D K K K C Y **Stop** L S T I **Stop** **Stop** F **Stop** K S Q S N S C V S **Stop** Y Met R H Q R S S S **Stop** E V Y L V D N

**Aligned TRH precursor peptide sequences in *P. lividus* and *S. puprupartus*:** TRH precursor sequences were aligned using Clustal Omega (<https://www.ebi.ac.uk/jdispatcher/msa/clustalo>). The annotation is based on (Rowe and Elphick, 2012; Van Sinay et al., 2017). In red is shown the predicted N-signal peptide. In green are highlighted the copies of the most abundant TRH form in TRH, which will give rise to the mature neuropeptide QYPGa. Our custom TRH antibody was specifically synthesized against this isoform. In Magenta are highlighted the copies of the second TRH form, QWPG, which would give rise to the mature QWPGa.

CLUSTAL O(1.2.4) multiple sequence alignment

Pl_TRH MKGTMVFYLWACILGYITWSGTALPTVLGKELVITEDDEPDIAGWDQGAEIPLRHHFWGD 60

Sp_TRH --------MWACILGYVTWGGAALPTILGKELVLSENDGPEIADWVQGKEIPLRNQYWGD 52

:*******:**.*:****:******::*:* *:**.* ** *****:::***

Pl_TRH IADAAEEEELDMLSPDSDKRQYPGGKRQYPGGKRQYPGGKRQYPGGKRQYPGGKRQFVGG 120

Sp_TRH VAEEEEEEELGMLSPDSEKRQYPGGKRQYPGGKRQYPGGKRQYPGGKRQFPAGKRQFVGG 112

:*: *****.******:*******************************:*.********

Pl_TRH ELIPSPELRQYPGGKRQYPGGKRQYPGGKRQYPGGKRQYPGGKRQYPGGKRSEGNQDLLP 180

Sp_TRH ELIPSPELRQWPGGKRQWPGGKRQWPGGKRQYPGGKRQYPGGKRQWPEV----------- 161

**********:******:******:********************:*

Pl_TRH MEIRQYPGGKRSEGDQDLLPMEIRQYPGGKRSEGDQDLLPMDGGKRQYPGGKRQWPGGKR 240

Sp_TRH --KRQYPGGKRSEDDQDLLPMEIRQY----------------------PGGKRQWPGGKR 197

**********.************ ************

Pl_TRH QYPGGKRQWPGGKRQFPGGKRQFVGGEAFEQEANINKRFVPEDETMDFLRLSQLYDTNEN 300

Sp_TRH QYPGGKRQYPGGKRQFPGGKRQFVGGEALEQESNINKRFAPEDDTMDFFRLSQLYDTNDN 257

********:*******************:***:******.***:****:*********:*

Pl_TRH LAADEEELAIEDLLDDIMVDTRPEMEEPMDMLLGDVDSEDALALDLSALLGNRTPINDW 359

Sp_TRH IVADEGELALEDLLDDIMVDTRPEFEDPRDLLLGNVDQEDVLALDLSALLGDRNPNNGW 316

:.*** ***:**************:*:* *:***:**.**.**********:*.* *.*


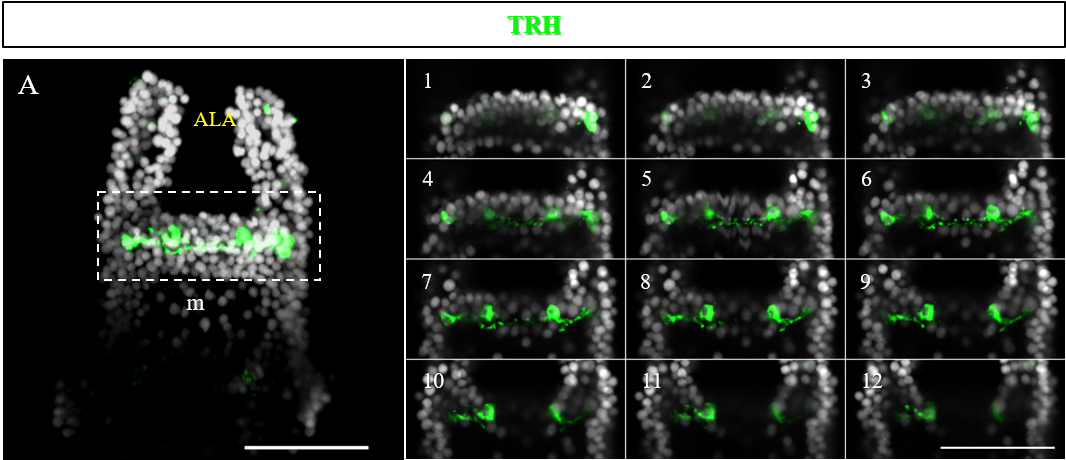


**Supplementary Figure 1.** Distribution of TRHergic cells from a ventral view. **(A)** Detail from Figure 2 K-L’. Numbers indicate the individual stacks.

Nuclei are labeled with DAPI (in white). All pictures are maximum projections of confocal images taken using a 25x water immersion objective.

m=mouth, ALA=Anterolateral arm, scale bars = 50 µm


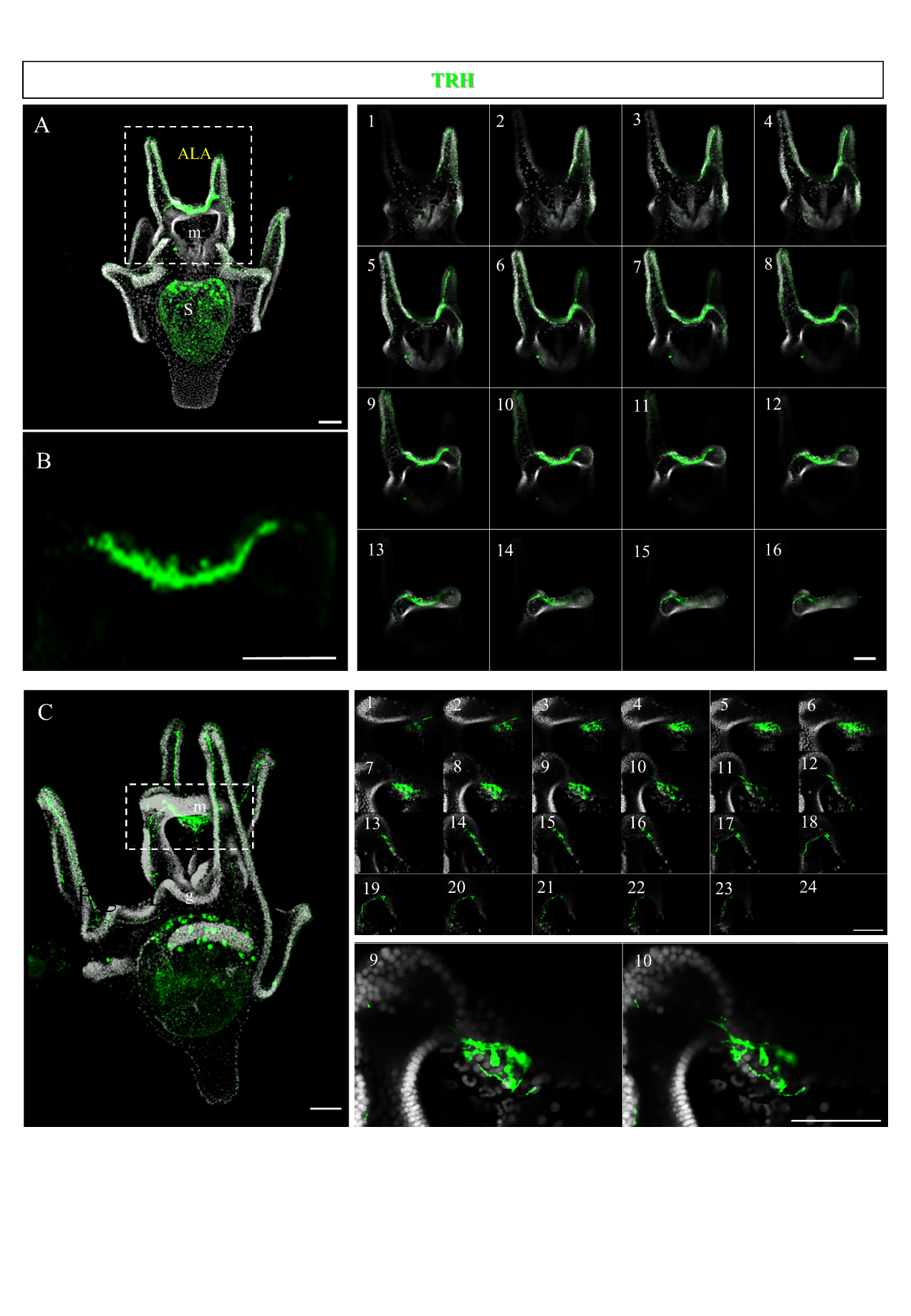


Supplementary Figure 2. Distribution of TRHergic cells in the oral hood of a sea urchin at 6-arm larva stage. (A,B) Details from Figure 3E-H highlighting the distribution of TRHergic cells in the oral hood and in the oral hood. (C) Detail of Figure 3I-L, a late 6-armed larva shows accumulation of TRHergic cell bodies in the upper-lip of the mouth. Many projections connect these cell bodies. Numbers indicate the individual stacks.

Nuclei are labeled with DAPI (in white). All pictures are maximum projections of confocal images taken using a 25x water immersion objective. m=mouth, s=stomach, ALA=Anterolateral arm, scale bars = 50 µm


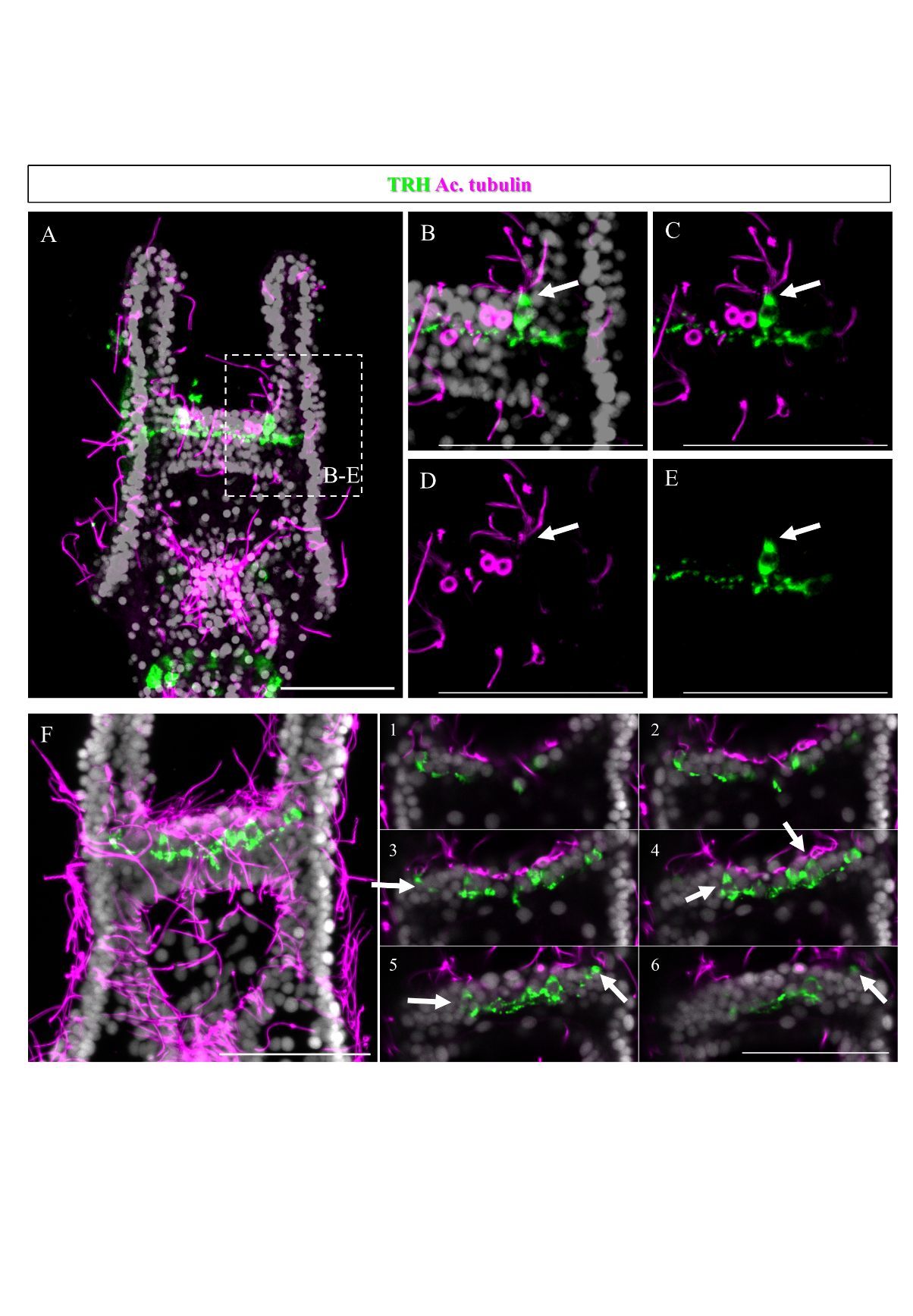


Supplementary Figure 3. TRHergic cells are ciliated cells also in *P. lividus*. (A-E) Detail from Figure 4 A-D showing that TRHergic cells are ciliated. (F) Detail from figure 4E-H confirm that also at later stages the TRHergic cells are ciliated. Moreover, in the stack 5the two TRHergic cells at the sides are connected by a set of axons and interneurons. Numbers indicate the individual stacks. White arrows highlight TRHergic cells which clearly have a cilium.

Nuclei are labeled with DAPI (in white). All pictures are maximum projections of confocal images taken using a 25x water immersion objective. m=mouth, s=stomach, ALA= Anterolateral arm, scale bars = 50 µm


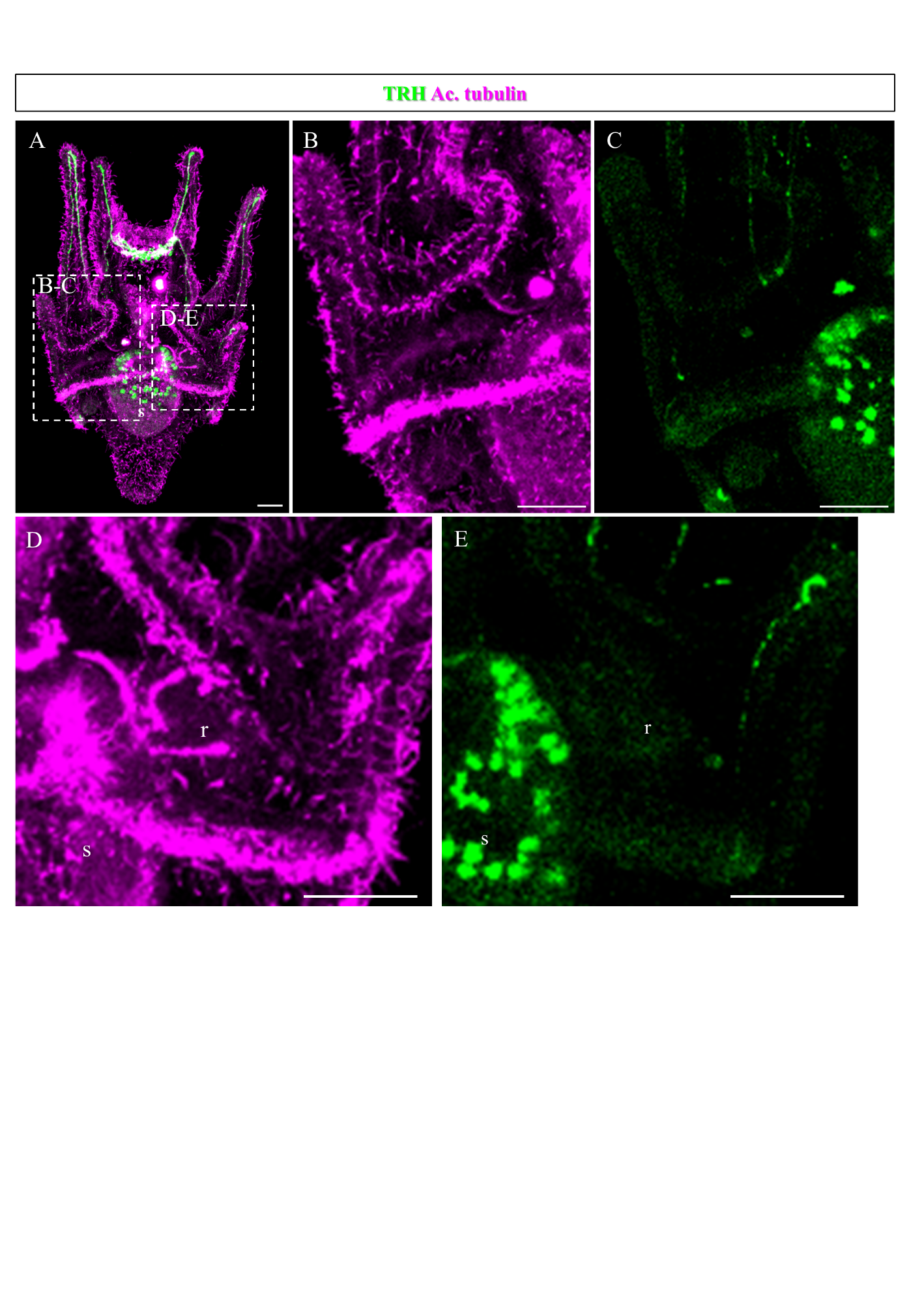


Supplementary Figure 4. Details of the double TRH/Acetylated tubulin immunostaining performed on *P. lividus* larvae at 6-armed pluteus stage. Detail from Figure 4Q-T. (B,C) Focus on the right side of the larva, showing no TRHergic axons innervating the eupaulettes. (D,E) Focus on the rudiment.

All pictures are maximum projections of confocal images taken using a 25x water immersion objective. r=rudiment, s=stomach, scale bars = 50 µm


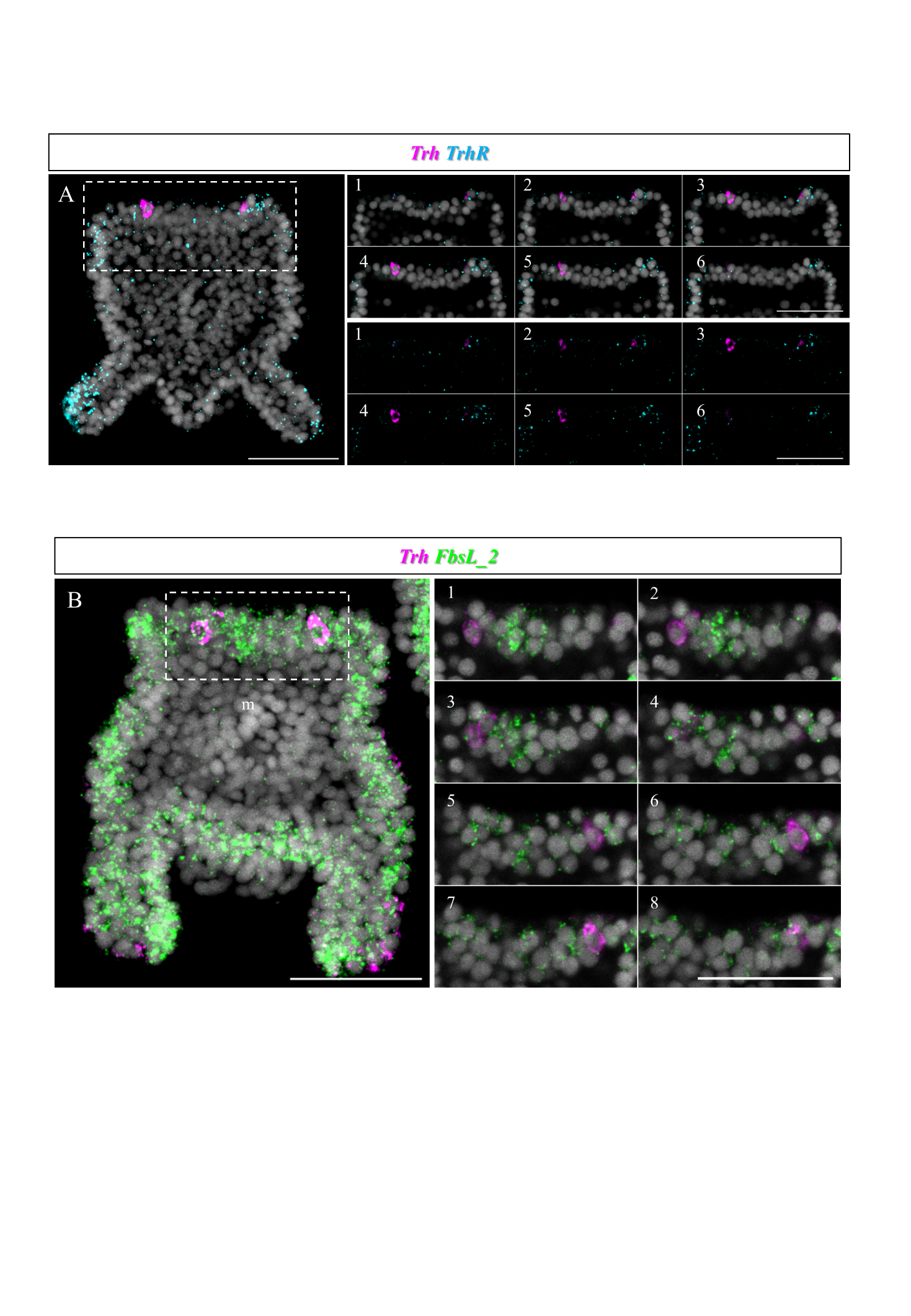


Supplementary Figure 5. *Trh* and *TrhR* are not coexpressed, as well as *Trh* and *FbsL_2*. (A) Detail from figure 8A,B. Despite the full projection seems to suggest that *Trh* and *TrhR* overlap, the individual stacks show no overlap between them. (B) Detail from Figure 8E-H. as in A, despite the full projection seems to suggest that *Trh* and *FbsL_2* overlap, the individual stacks show no overlap between them.

Numbers indicate the individual stacks.Nuclei are labeled with DAPI (in white). All pictures are maximum projections of confocal pictures taken using a 25x water immersion objective. m=mouth, scale bars = 50 µm

| Gene | Primer Forward | Primer Reverse | Labelling | Reference and notes |
| --- | --- | --- | --- | --- |
| Trh | TGTGGGACTTAGCAAGAGGG | ACCAGTCATTGATCGGGGTT | Fluo |  |
| Sp-TrhR | CGAAGGGCTGCTAAGATCAC | CGCTAATTGGCCGCAGTAG | DIG | Clones gifted by Oliveri’s lab (UCL) (Wood, 2020) |
| Sp-Tph | \| ATCGAATCGAGAAAGGCTCA \| \| --- \| | GGTCAATTCGTCTCGGACAT | DIG | (Paganos et al., 2021) |
| Sp-FbsL_2 | TTTCACACGCTTCATCATCC | TGATAGCTGCTGCTCCACAT | DIG | (Paganos et al., 2021) |
| Opsin3.1 | TCGAAGCTTCAACCCGAGTA | GATCCGCTCCTTGTCGTAGA | DIG |  |
| Gi | AATAGAGGTCGTCGGTAGCG | GCACTGAGAGCCACACAAAA | DIG |  |
| Chat | GCGAGCCCAGTCAATATGTG | CCCAGAGCAAATCATGTCCG | DIG |  |
| Sp-Ddc | CCACCGATGACAAAGGTTCT | CCAGGCATACGTCATGTGTC | DIG | (Paganos et al., 2021) |

Supplementary Table 1. Primers used to clone the gene of interests. For the the *Sp-* genes, probes were produced against the *S. purpuratus* mRNA and used to perform in situ in *P. lividus* after being checked for their specificity by blasting Sp/probe sequence against *P. lividus* assembly in <https://genome-euro.ucsc.edu/cgi-bin/hgBlat?hgsid=315448589_FK6p7kpXBjgLnREHayOvp8cagdzO&command=start>.

| Antigen | Dilution | Species | Reference |
| --- | --- | --- | --- |
| TRH | 1:400 | Rabbit | (Wood, 2020; Cocurullo et al., 2023) |
| Acetylated tubulin | 1:250 | Mouse | Sigma Aldrich |
| Serotonin | 1:1000 | Rat | Santa Cruz Biotechnology |
| 1E11 | undiluted | Mouse | (gift from Dr. Robert Burke |
| β-Tubulin | 1:250 | Mouse | Sigma Aldrich |

Supplementary Table 2. Antibodies dilutions and details.
